# Sequential Extraction, Characterization, and Analysis of Pumpkin Polysaccharides for Their Hypoglycemic Activities and Effects on Gut Microbiota in Mice

**DOI:** 10.3389/fnut.2021.769181

**Published:** 2021-11-03

**Authors:** Hui-qing Wu, Zhi-li Ma, De-xin Zhang, Ping Wu, Yuan-hua Guo, Fang Yang, De-yuan Li

**Affiliations:** Wuhan Functional Food Engineering and Technology Research Center, School of Laboratory Medicine, Hubei University of Chinese Medicine, Wuhan, China

**Keywords:** pumpkin polysaccharides, sequential extraction and purification, structure characterization, antioxidant activity, hypoglycemic activity, gut microbiota

## Abstract

This study aimed to extract polysaccharides from pumpkin, characterize the structures of four of them, and evaluate their *in vitro* antioxidant and hypoglycemic activities. Additionally, an animal model of type 2 diabetes mellitus (T2DM) was established and used to determine their hypoglycemic and hypolipidemic effects *in vivo*, and the underlying mechanisms related to the regulation of gut microbiota. Water-extracted crude pumpkin polysaccharides (W-CPPs), water extraction and alcohol precipitation crude pumpkin polysaccharides (WA-CPPs), deproteinized pumpkin polysaccharides (DPPs), and refined pumpkin polysaccharides (RPPs) were sequentially extracted and purified from pumpkin powder by hot water extraction, water extraction, and alcohol precipitation, deproteinization and DEAE-52 cellulose gel column, respectively. The extraction and purification methods had significant influence on the extraction yield, physicochemical properties, and *in vitro* antioxidant and hypoglycemic activities. W-CCP and RPPs had a significant positive free radical-scavenging capacities and inhibitory activities on α-glucosidase and α-amylase. RPP-3 not only inhibited the uptake of glucose in Caco-2 monolayer but also promoted the excretion of glucose, while RPP-2 had no inhibitory effect. Animal experiment results showed that W-CPP treatment significantly improved the T2DM symptoms in mice, which included lowering of fasting blood glucose (FBG), reducing insulin resistance (IR), and lowering of blood lipid levels. It increased the diversity of intestinal flora and reduced the harmful flora of model mice, which included *Clostridium, Thermoanaerobe, Symbiotic bacteria, Deinococcus, Vibrio haematococcus, Proteus gamma*, and *Corio*. At the family level, W-CPP (1,200 mg/kg) treatment significantly reduced the abundance of *Erysipelotrichaceae*, and the *Akkermanaceae* of *Verrucobacterium* became a biomarker. Pumpkin polysaccharides reshaped the intestinal flora by reducing *Erysipelotrichaceae* and increasing *Akkermansia* abundance, thereby improving blood glucose and lipid metabolism in the T2DM mice. Our results suggest that W-CCP and RPP-3 possess strong antioxidant and hypoglycemic activities, and are potential candidates for food additives or natural medicines.

## Introduction

Diabetes mellitus is a metabolic disease characterized by chronic hyperglycemia, which is caused by a variety of causes. Due to defective insulin secretion or impairment of insulin actions, long-term high blood sugar level can cause complications in various tissues, leading to chronic damage and dysfunction, especially the kidneys, eyes, and blood vessels (1). Type 1 diabetes mellitus (T1DM) and type 2 diabetes mellitus (T2DM) are the common types. T1DM is only about 10% and often develops in childhood and adolescence, because the destruction of pancreatic β-cell leads to an absolute deficiency or a significant decrease in insulin. Because of insulin resistance, progressive insufficient insulin secretion, or both, T2DM is relatively common and accounts for around 90% of all diabetes cases worldwide (2). According to the latest International Diabetes Federation (IDF) Diabetes Atlas, the number of people aged 20–79 with DM in 2019 was about 463 million, while there were 351.7 million in the working group aged 20–64, and the prevalence of DM is climbing year by year. In China, DM is now one of the four major chronic diseases, and chronic non-communicable diseases have become the leading cause of death and disease burden among the Chinese population, showing that DM is a global public health problem (3). Diet is the first risk factor for mortality and life expectancy loss, which fully illustrates the importance of dietary nutrition.

At present, in addition to the control of energy intake and exercise, drug therapy is required. Oral hypoglycemic drugs include sulfonylureas, metformin hydrochloride, α-glucosidase, sodium-glucose cotransporter-2 (SGLT2) inhibitors, dipeptidyl peptidase-4 (DPP-4) inhibitors, and glucagon-like peptide-1 (GLP-1) analogs (4). These drugs are effective in controlling hyperglycemia, but they also have significant side effects, such as hypoglycemia, gastrointestinal disorders, and weight gain, and they may cause drug dependence in patients (5). Therefore, it is important to find effective alternatives with low toxicity and without side effects. Dietary polysaccharides have been considered by investigators because of their low toxicity and numerous pharmacological activates, such as enhance immunity, anti-tumor, anti-mutation, lowering blood pressure, lowering blood fat, lowering blood sugar, anti-viral, anti-oxidation, anti-radiation, anti-coagulation, anti-thrombosis, and anti-aging (6). Now, the hypoglycemic activity mechanism of dietary plant polysaccharides is mainly as follows: (1) promote insulin secretion, which mainly promotes insulin secretion through anti-oxidation, anti-inflammatory, hypolipidemic, and hypoglycemic protection of the structure and function of islet β-cells; (2) enhance insulin sensitivity and improve insulin resistance; (3) regulate key enzyme activity, and promote sugar absorption, utilization, and metabolism; (4) regulate the signal pathway, activate the PI3K/Akt pathway, and, finally, regulate the ERK/JNK/MAPK pathway (5, 6) plant polysaccharides also have a regulatory effect on intestinal microbes (7). Pumpkin (*Cucurbita moschata*) is one of the most popular vegetables in the world, and it contains carotenoids, pectin, dietary fiber, mineral elements, polysaccharides, para-aminobenzoic acid, sterols, proteins, and peptides. At present, researchers have shown that pumpkin polysaccharides can be used as potential hypoglycemic substances and have certain therapeutic effects on diabetes (4–8), antioxidant activity (4, 9–12), lowering plasma lipids (8), improving immunity (9), and anti-cancer (13). Currently, studies on *in vitro* hypoglycemic activity in pumpkin polysaccharides mainly focus on radical-scavenging ability assay, plasma iron reduction ability assay, α-glucosidase and α-amylase inhibition assay, bile acid binding ability assay, and pancreatic islet β-cell damage protective ability assay (4, 9, 11–13). Among these studies, the different extraction methods had different *in vitro* hypoglycemia activity, which is related to its monosaccharide types and molar ratios of the pumpkin polysaccharides. Moreover, the *in vitro* hypoglycemia activity results had no uniform method for quantitative comparison. The human epithelial Caco-2 cell monolayer model can be used to quantify the absorption of glucose and as a supplement to evaluate the *in vitro* hypoglycemia activity (14).

*In vivo* research mainly focused on animal experiments. Current studies have shown that the regulatory mechanism of some polysaccharides in the body is closely related to intestinal microbes. Yan et al. showed that the FBG and insulin of T2DM rats were effectively controlled after the administration of total polysaccharides of polygonatum (0.1 g/kg) for 56 days. At the same time, it improved the ratio of gut microbes in rats by reducing the abundance of *Bacteroides* and *Proteobacteria* and increasing the abundance of *Firmicutes*. Polygonatum polysaccharides could also reduce the production of short-chain fatty acids, such as acetic acid, propionic acid, and butyric acid (15). In the study on ophiopogon japonicus polysaccharides, giving 300 mg/kg ophiopogon japonicus polysaccharides to obese mice for 12 weeks could reduce the ratio of *Firmicutes/Bacteroides* and restore the composition of the mouse intestinal flora to a normal state. At the same time, it increased the production of taurine, short-chain fatty acids, and their metabolites, thereby reducing blood lipids and blood sugar in mice. In addition, the indigestible ophiopogon japonicus polysaccharide degraded and utilized by the gut microbiota, and then absorbed and utilized by the host, thereby exerting the effects of weight loss, energy metabolism, and immune system enhancement (16). The research conducted by Liu et al. showed that during the treatment of T2DM in rats, pumpkin polysaccharide (1,000 mg/kg) changed the structure of gut microbiota and selectively enriched key species of *Bacteroidetes, Prevotella, Deltaproteobacteria, Oscillospira, Veillonellaceae, Phascolarctobacterium, Sutterella*, and *Bilophila*, wihch is similar with the effect of metformin of enriching the gut microbiota that produces short-chain fatty acids (7). Phytonutrients could affect the absorption and metabolism of intestinal glycolipids by regulating the growth of intestinal flora, and, in turn, the blood sugar of the body and lipids (17). The intestinal flora plays an important role in assisting digestion, host defense, and the activation of the immune system. In turn, the host environment and immune system can affect the intestinal flora. These phytonutrients can directly stimulate the intestinal flora and provide nutrients for probiotics. On the other hand, some nutrients need to be decomposed and transported by the intestinal microbial enzyme system to exert their unique physiological activities. At present, the possible mechanisms of hypoglycemia action by intestinal flora include short-chain fatty acid theory, bile acid theory, and endotoxin theory (18, 19). However, there are relatively few studies focusing on the causal relationship between polysaccharide-induced changes in the intestinal microbiome and hypoglycemic effects. Therefore, whether pumpkin polysaccharides, in addition to improving insulin resistance and reducing some biochemical indicators, can affect blood sugar and blood lipids through the regulation of intestinal flora and its metabolites is still unknown. This is a very meaningful study to promote the development of the mutual regulation of the intestinal microbiome, polysaccharides, and hypoglycemic effects. In this study, four pumpkin polysaccharides were separated and purified to evaluate their *in vitro* antioxidant activities, α-glucosidase and α-amylase inhibitory activities, and uptake and transport assay of glucose inhibitory activities. Furthermore, we constructed a T2DM mouse model to explore the hypoglycemic and hypolipidemic effects of pumpkin polysaccharides, as well as the mechanism related to the regulation of gut microbiota. The highly active polysaccharide components were suitable for further research, providing a theoretical basis for the future development of a functional food additive or natural medicine candidate.

## Materials and Methods

### Materials

Pumpkin powder (DRN-1300) was acquired from Powdery-Hubei Health Industry Co., Ltd., (Jingmen, China). Pumpkins were selected, peeled, and cut into pieces. With seeds and flesh removed, the pumpkin meat was broken into pulp, ground, and homogenized, and then sterilized at a high temperature instantaneously, then spray-dried instantaneously at a temperature of 190°C to obtain pumpkin powder. The pumpkin powder on a dry weight basis (%, w/w) was presented as follows: carbohydrate 74.1%, protein 8.4%, lipids 2.2%, moisture 4.3%, and ash 8.4%. Tris, DEAE-52 cellulose, trifluoroacetic acid (TFA), bovine serum albumin (BSA), p-nitrophenyl-α-D glucopyranoside (pNPG), 1,1-diphenyl-2-picryl-hydrazyl (DPPH), 2,20-azino-bis-3-ethylbenzthiazoline-6-sulphonic acid (ABTS^+^), acarbose, L-ascorbic acid (VC), α-glucosidase, α-amylase, glucose oxidase assay kit, and monosaccharide standards, rhamnose (Rha), arabinose (Ara), xylose (Xyl), ribose (Rib), fructose (Fru), mannose (Man), glucose (Glc), galactose (Gal), fucose (Fuc), and inositol were purchased from Shanghai Yuanye Bio-Technology Co., Ltd., (Shanghai, China). Triglycerides (TG), total cholesterol (TC), low-density lipoprotein cholesterol (LDL-C), high-density lipoprotein cholesterol (HDL-C), and glycosylated serum protein (GSP) kits were purchased from Nanjing Jiancheng Institute of Biological Engineering (Nanjing, China). Insulin determination kit (ELISA; rat insulin ELISA kit) was purchased from Hualian Branch Biotechnology (Wuhan, China); Streptozotocin (STZ, 98%) was purchased from Sigma-Aldrich (Bornem, Belgium). Roche blood glucose meter and Accu-chekactive test strips were purchased from Roche Diabetes Care GmbH (Basel, Switzerland).

Caco-2 cells were purchased from American Tissue Culture Collection (Rockville, United States). C57BL/6J male mice (6 weeks old) were purchased from Liaoning Changsheng Biotechnology Co., Ltd., (Liaoning, China) with license number SCXK (Liao) 2020-0001, and the animal quality certificate number was 210726201100350774. Dulbecco's modified Eagle medium (DMEM, SH30022.01), 0.25% trypsin with ethylenediaminetetraacetic acid (EDTA), fetal bovine serum (FBS), 100 × penicillin and streptomycin, and 100 × non-essential amino acids were obtained from Thermo Scientific HyClone (Logan, United States). Twelve-well cell culture plates and transwell permeable polycarbonate inserts (0.4 μm) were purchased from Corning Costar (New York, United States). Lucifer yellow, HEPES sodium salt 99%, non-essential amino acids, dimethyl sulfoxide (DMSO), 3-(4, 5-dimethylthiazole-2-yl)-2, 5-diphenyltetrazolium bromide (MTT) and Hank's Balanced Salt Solution (HBSS) buffer constituents were obtained from Sigma-Aldrich (Bornem, Belgium). Phusion® High-Fidelity PCR Master Mix was brought from New England Biolabs (Massachusetts, United States). Primers were provided by Sango Biotech (Shanghai, China). Other reagents were from Sinopharm Chemical Reagent Co., Ltd., (Shanghai, China).

### Pumpkin Polysaccharide Sequential Extraction and Purification

Crude pumpkin polysaccharides were prepared according to the method of Chen et al. after the optimization experiments (9). Pumpkin powder was added with distilled water at 60°C according to the material-to-liquid ratio of 1:30 and extracted in a water bath at 80°C for 4 h. The supernatant was collected after centrifugation at 2,500 × g for 15 min. A part of the supernatant was purified by nanofiltration at 40°C and 1 MPa, decolorized with activated carbon, and freeze dried to obtain water-extracted crude pumpkin polysaccharides (W-CPPs). The remaining supernatant was concentrated by distillation under reduced pressure until about 1/3 of the original volume, and then sonicated for 30 min. Anhydrous ethanol was added to make the ethanol volume fraction reach 70%. The mixture was allowed to stand at 4°C for 12 h to collect the precipitate. The above process was repeated twice. The precipitate was washed twice with ethanol and acetone, volatilized, decolorized, and freeze-dried to obtain water extraction and alcohol precipitation crude pumpkin polysaccharides (WA-CPPs).

Deproteinized pumpkin polysaccharides were prepared according to the method of Liu et al. (20). A WA-CPP solution (25 mg/ml) was mixed with 25% (w/w) Sevage reagent under continuous stirring at 150 r/min and 25°C for 15 min in a shaking incubator, while the Sevage reagent was pre-prepared by n-butanol: chloroform with a volume ratio of 1:4. After centrifugation (4,000 r/min, 30 min), the lower organic phase and deformed protein at the junction were removed. The above procedures were repeated three times, and ethanol was precipitated, concentrated, and freeze-dried to obtain deproteinized pumpkin polysaccharides (DPPs). Refined pumpkin polysaccharides (RPPs) were prepared as described with some modifications (21). DPPs were dissolved in deionized water to a 10-mg/ml solution using ultrasound. After filtration with a 0.45-um pore membrane, the DEAE-52 cellulose gel column (2.6 × 50 cm) was used for purification. The DEAE-52 cellulose gel column is eluted with distilled water first, and then eluted with gradient NaCl solution (0.1, 0.3, and 0.5 mol/L). Each fraction of 10 ml was collected at a flow rate of 1 ml/min. The total carbohydrate content was measured by phenol-sulfuric acid method at 490 nm, and then polysaccharide-containing tubes were collected and concentrated with a rotary vacuum evaporator. The collected fractions were dialyzed (MWCO: 3.5 kDa) against distilled water for 48 h, and freeze-dried to obtain four refined polysaccharide fractions (RPP-1, RPP-2, RPP-3, RPP-4), which were stored at −80°C for later use.

### Physicochemical Properties of Pumpkin Polysaccharides

The extraction yield (%) of crude pumpkin polysaccharides was estimated according to the following equation: extraction yield (%, w/w) = [weight of the crude dried pumpkin polysaccharides (g)/weight of the dried powder (g)] × 100%. The extraction yield (%) of RPPs was calculated as [weight of the RPPs (g)/weight of the DPPs (g)] × 100%. The total carbohydrate content was measured at 490 nm by phenol-sulfuric acid method (22), and polysaccharide retention rate of DPPs was calculated as [weight of the DPPs (g)/weight of the WA-CCPs (g)] × 100%. The Bradford method with BSA as a standard was used to determine the protein content (23), and the protein removal rate of DPPs was calculated as = [(protein content of the WA-CCP (μg/ml) – protein content of the DPPs (μg/ml))/ protein content of the WA-CCPs (μg/ml)] × 100%. A UV-vis spectrophotometer was used to scan the RPPs (1 mg/ml) in the range of 200–800 nm. The sulfuric acid-carbazole method was used to determine the uronic acid content in the polysaccharide samples. The Folin-Ciocalteu assay with gallic acid as a standard was used to determine the total phenolic content. The iodine-potassium iodide reaction was used to detect polysaccharide side chain and branch structures. RPPs (2 mg) were added with 1.2 ml of the iodine-potassium iodide reagent (0.2% potassium iodide and 0.02% iodine), and the mixture was then scanned with the UV-Vis spectrophotometer in the wavelength range. The Congo red test can determine whether a polysaccharide sample has a triple helix structure (24). The Fehling and ninhydrin test was performed to detect the presence of reducing sugars, amino acids, and peptides.

### Monosaccharide Composition Analysis

The monosaccharide composition of RPPs were analyzed with Agilent 7890 gas chromatogram (GC; Agilent Technologies, Inc., United States) according to the method of Wang et al. (25). RPPs (10 mg) were hydrolyzed with 2 mol/L TFA (5 ml) for 3 h at 120°C. After evaporating the TFA under reduced pressure, the hydrolysate was dissolved in 2–3 ml methanol, and then evaporated to dryness, and the process was repeated thrice. Then, hydroxylamine hydrochloride (10 mg), inositol (internal standard, 2 mg), and pyridine (1 ml) were added in that sequence, and the reaction was allowed to proceed for 30 min at 90°C. After cooling, acetic anhydride (1 ml) was added for 30 min at 90°C, and then distilled water (1 ml) was added to shake well. Finally, the mixture was extracted thrice with chloroform, and the chloroform layer was taken for GC analysis. The operation was performed using the following conditions: H_2_: 16 ml·min^−1^, air:150 ml·min^−1^, N_2_: 20 ml·min^−1^, injection temperature: 270°C, detector temperature: 270°C, column temperature programmed from 150 to 190°C at 10°C·min^−1^, held for 5 min at 190°C, then increased to 220°C at 2°C·min^−1^, and finally held for 10 min at 210°C.

### Fourier Transform Infrared Spectroscopic Analysis

The primary structure of polysaccharides can be determined by FT-IR. The KBr tablet method was used for the infrared spectroscopy analysis. The dried RPPs were ground with KBr powder and then pressed into complete and transparent sheets for measurement in the range of 400–4,000 cm^−1^ using a Nicolet iS50 FT-IR spectrometer (Thermo Fisher Ltd., United States).

### Scanning Electron Microscopy Observation

A scanning electron microscope (SEM) was used to observe the surface microstructure of the freeze-dried sample RPPs. The samples were sprayed with gold under vacuum, and SEM images were observed on a JSM-6510LV scanning electron microscope (JEOL Ltd., Japan). Photos, under different magnifications, were taken for analysis.

### Determination of *in vitro* Antioxidant Activity

Four chemical assays to determine hydroxyl (·OH), DPPH, superoxide anion (O_2_·^−^), and ABTS^+^ radical-scavenging ability were conducted to evaluate the *in vitro* antioxidant activities of the pumpkin polysaccharides. For all the assays, W-CCPs, WA-CCPs, and DPPs were prepared at the polysaccharide samples the polysaccharide samples concentrations (2–10 mg/ml), while the concentration of RPPs ranged from 0.2 to 1 mg/ml. Meanwhile, the positive control and blank in the antioxidant reactions were V_C_ and distilled water, respectively. According to the method of Liu et al. (26), the OH and O_2_·^−^ radical-scavenging abilities were measured before recording absorbance with the salicylic acid method at 510-nm wavelength and pyrogallol at 510-nm wavelength, respectively. The scavenging capacity against DPPH and ABTS^+^ free radicals was assessed with radicals reacting in the dark with samples for 10 min at 519 and 734 nm by spectrophotometry, respectively, and as described by Chen, Ma, and Kitts (27). The absorbance was measured using a UV-100 spectrophotometer (Shanghai Aoyi Analytical Instrument Co., Ltd., China) operated at room temperature.

### Determination of *in vitro α*-Glucosidase and α-Amylase Inhibitory Activities

The hypoglycemic activities of the four pumpkin polysaccharides *in vitro* were measured by evaluating the α-glucosidase and α-amylase inhibitory capacities (28). For all the assays, W-CCPs, WA-CCPs, and DPPs were prepared at different concentrations (2–10 mg/ml), while the concentration of RPPs ranged from 0.2 to 1 mg/ml. Then, 20 μl of 5 U/ml α-glucosidase and 100 μl of 2.5 mmol/L pNPG reaction, the 200 μL of 12.5 U/ml α-amylase and 1% (w/w) starch reaction that occurred at 37°C for 10 and 15 min in the presence of the different samples, then were separately detected at 405 nm wavelength and 540 nm by spectrophotometry to evaluating the α-glucosidase and α-amylase inhibitory capacities, respectively. Meanwhile, the positive control and blank in the enzymatic reactions were acarbose and distilled water, respectively. The absorbance was measured with Synergy ™ 2 Multi-Mode Microplate Reader (Biotek Instruments Co., Ltd., United States) operated at room temperature.

### Glucose Uptake and Transport Experiments

Human colon cancer cells (Caco-2) were cultured according to Tavelin (29), with some modifications. Caco-2 cells with passage numbers 31–41 were cultured in a 25-cm^2^ cell culture flask at 37°C in a high-glycemic DMEM medium containing 5% CO_2_, and the DMEM contains 20% (v/v) heat-killed FBS, 1% non-essential amino acids, 1% L-glutamine, 20 mmol/L HEPES, 100 μg/ml streptomycin, and 100 U/ml penicillin (pH 7.4). The cells were seeded in a polycarbonate microporous membrane (0.4 μm pore size, 12 mm diameter) inserted into a transwell at a density of 1.25 × 10^4^ cells/cm^2^ and cultured for 21 days, once they reached 80% confluence. In the glucose transport experiment, the observation and measurement methods of the establishment of the Caco-2 monolayer model, alkaline phosphatase activity, integrity of the differentiated cells in each monolayer, and the transepithelial electrical resistance (TEER) referred to the protocol described in detail previously (30, 31). The cytotoxicity and safe concentration range of pumpkin polysaccharides RPP-1, RPP-2, RPP-3, and RPP-4 in the Caco-2 cells were determined by the MTT assay at 570-nm absorbance.

The glucose uptake experiment was examined with the method of Yang et al. (30, 31) and Zhu et al. (5). The pumpkin polysaccharide RPP solutions (0, 0.2, 0.4, 0.6, and 0.8 mg/ml) with or without 50 mmol/L glucose in HBSS solution were added to 12-well plates. The cells were then incubated in the medium for 60 and 120 min. In the blank group and control group, the Caco-2 cells were incubated with a culture media and culture media with 50 mmol/L glucose, respectively. At the end of the incubation, the cells were lysed and centrifuged to obtain the supernatant, fats were removed with Sevage reagent, and then the volume was adjusted to 1 ml with deionized water to detect the glucose content with a glucose oxidase assay kit (Shanghai Yuanye Bio-Technology Co., Ltd.,) at 505 nm by spectrophotometry. The glucose uptake inhibition rate was calculated as in Eq. (1):


(1) $$\begin{array}{l} \text{Glucose uptake inhibitionrate }\left(\%\right)\\ \;=\left({\text{A}}_{\text{C}}-{\text{A}}_{\text{S}}\right)/\left({\text{A}}_{\text{C}}-{\text{A}}_{\text{B}}\right)\times 100 \end{array}$$

where AS, AC, and AB are the absorbance of the sample, control, and blank group, respectively.

The glucose transport experiment for the apparent permeation rate across the Caco-2 cells used the previous protocol (13, 30, 31). To perform the transport experiments in the apical-to-basolateral (AP-BL, absorptive) direction, the pumpkin polysaccharide RPP solutions with 50 mmol/L glucose in HBSS solution (0.6 mg/ml in 0.5 ml) were added to inserts of 12-well transwell, where cells were grown for 21 days, and 20 mmol/L HEPES with 4% BSA in the HBSS solution (pH 7.4, 1.5 ml) was added into 12-well transwell BL side. In the basolateral chamber, BSA had a final concentration of 4% to mimic the physiological situation. Then, 100 μl samples were collected from the receiving chamber at 0, 30, 60, 90, and 120 min. In order to maintain a constant volume, an equal volume of the blank transport medium was added after each sampling. For the transport of the basolateral-to-apical (BL-AP, secretory) direction, the added solutions on both sides of the above 12-well transwell were interchanged, and the other follow-up operations were the same as above.

The cumulative amount of transported glucose can be calculated with the apparent permeability [*P*_app_ (in cm/s)] in Equation (2), where *dQ/dt* means constant flow (dpm/s), *A* is the filter membrane surface (cm^2^), and *C*_0_ is the initial glucose concentration of the side supply (dpm/cm^3^). The cumulative fraction transported (FAcum) of glucose can be calculated as in Equation (3), and it indicates that the cumulative amount of material being transported gradually increased.

The variables *V* and *C* represent the volume and concentration of the supply solution (index D) or receiving solution (index R). Then, *f* is the sample replacement factor with the formula *f* = *1* – *V*_*S*_*/V*_*R*_, in which *V*_*S*_is the sampling volume. When the receiver concentration exceeds 10% of the supplying liquid concentration, *P*_app_ can be defined as in Equation (4), where *M* is the total mass of the system balance, *C*_*R*0_ is the initial concentration of glucose in the receiving chamber, and *C*_*R*_*(t)* is the concentration of glucose at the time *t*. The uptake ratio (UR) is the ratio of absorption permeability to secretion permeability, which can be expressed as (*P*_app, AP−BL_/*P*_app, BL−AP_).


(2) $$\begin{array}{l} p_{app}=\left(\frac{dQ}{dt}\right)\times \left(\frac{1}{AC_{0}}\right) \end{array}$$

(3) $$\begin{array}{l} FAcum=\frac{1}{A}\overset{i}{\underset{k=1}{\sum }}\frac{\left[C_{R}\left(t_{K}\right)-f\times C_{R}\left(t_{K-1}\right)\right]\times V_{R}}{\left[C_{D}\left(t_{K-1}\right)+C_{D}\left(t_{K}\right)\right]/2} \end{array}$$

(4) $$\begin{array}{l} C_{R}\left(t\right)=\left(\frac{M_{tot}}{V_{D}+V_{R}}\right)\\ \;+\left(CR_{0}-\frac{M_{tot}}{V_{D}+V_{R}}\right)\times e^{-P_{app}\times A\times \left(\frac{1}{V_{R}}+\frac{1}{V_{D}}\right)\times t} \end{array}$$

### Animal Experiment and Induction of Experimental Diabetes

All the mice were housed in the animal facility with stable moisture (50 ± 5%), dark-light cycle (12/12 h), and constant temperature (23 ± 2°C). After 1 week of accommodation, 84 mice were randomly divided into two groups: normal chow diet (NCD) and high-fat diet (HFD). The NCD and HFD groups were then fed for 4 weeks. After 28 days, the HFD mice were injected intraperitoneally with 200 mg/kg STZ to induce T2DM diabetes after a 12-h starvation period. Three hours after injection, the starved mice were fed with 4% glucose solution to prevent hypoglycemia. After 7 days, the mice were fasted again for 12 h, and blood glucose was quantified using a glucometer (Roche Diabetes Care GmbH). Mice with stable FBG of between 11.1 and 33.3 mmol/L for 2 weeks were chosen for further experiment (32). This animal experiment had passed the animal ethics review of the Animal Center of Hubei University of Traditional Chinese Medicine, with license number SCXK (E) 2017-0067. Then, the mice were separated randomly into the following four groups (*n* = 8/group): control group (non-diabetic rats, gavage with saline), model group (diabetic rats, gavage with saline), pumpkin polysaccharide low-dose group (PPS.L) (diabetic rats, gavage with W-CCPs at 600 mg/kg b.w.), and pumpkin polysaccharide high-dose group (PPS.H) (gavage with W-CCPs at 1,200 mg/kg b.w.). The four groups were treated by gavage for 28 days. The body weight and FBG levels were determined from tails with a Roche blood glucose meter weekly. After 28 days, all the mice were fasted for 12 h, and blood samples were collected and centrifuged to gain plasma. The plasma lipids, namely, TG, TC, LDL-C, HDL-C, and GSP, were evaluated using an automatic biochemical analyzer (Shenzhen Lei Du Life Technology Co., Ltd., Shenzhen, China). INS was evaluated by enzyme-linked immunoassay using a rat insulin ELISA kit. The cecal contents were taken out in a sterile state, mixed well, and stored in a refrigerator at −80°C for subsequent 16S rDNA microbial community analysis.

### 16S rDNA Microbial Community Analysis

The genomic DNA of the cecal contents was extracted with the CTAB method. Then, the purity and concentration of the DNA were detected by agarose gel electrophoresis. An appropriate amount of sample DNA was placed in a centrifuge tube, and the sample was diluted with sterile water to 1 ng/μl for PCR amplification. 16S rRNA genes of distinct regions (16S V3–V4) were amplified using a specific primer with a barcode. Next, the qualified PCR products were purified with magnetic beads and quantified by enzyme label, and then the samples were mixed in equal amounts according to the concentration of the PCR products. After fully mixing, the PCR products were detected by 2% agarose gel electrophoresis, and the target bands were recovered using a gel recovery kit from Qiagen company. TruSeq® DNA PCR-Free Sample Preparation Kit (Illumina, United States) was used to construct the library, which was assessed on the Agilent Bioanalyzer 2100 system. Finally, TruSeq® DNA PCR-Free Sample Preparation Kit was used to construct the library and quantified by Qubit and Q-PCR. After the library was qualified, the NovaSeq6000 was used for sequencing. The sequencing data were processed as follows: first, the quality control process for tags of Qiime (V1.9.1 http://qiime.org/scripts/split_libraries_fastq.html) was applied to the FLASH (V1.2.7, http://ccb.jhu.edu/software/FLASH/) software that processed the data. The tags were compared with the reference database (SUCHIME, http://www.drive5.com/usearch/manual/uchime_algo.html) to detect chimera sequences, and then the chimera sequences were removed. Then, the effective tags were finally obtained (33–35). Second, operational taxonomic unit (OTU) clustering and species annotation were performed. The Uparse software (v7.0.1001, http://www.drive5.com/uparse/) was applied to cluster all the effective tags of all the samples. By default, the sequence was clustered into OTUs with 97% identity. The Mothur method and the SSUrRNA database of Silva138 (http://www.arb-silva.de/) were used to perform species annotation analysis (set threshold 0.8–1). According to the relative abundance of species at each classification level in the OUT table, the R software (v2.15.3) was used to draw histograms and heat maps. Third, alpha diversity is evaluated by QIIME, including five indicators of species as Chao1, Ace, Shannon, Simpson, and Dominance to analyze the complexity of sample species diversity, and use R software to plot and display. Fourth, beta diversity is a comparative analysis of microbial community composition of different samples. QIIME was used to calculate unifrac distance and construct a UPGMA sample clustering tree. PCA, PCoA, and NMDS diagrams are drawn with the R software. Fifth, the LEfSe software was used to perform LEfSe analysis, and the default LDA Score was set to 4 to find out the biomarkers of each group. The raw sequencing data generated in this study have been stored in NCBI SRA with the BioProject ID PRJNA760239.

### Statistical Analysis

All the experiments were repeated three times, the results were expressed as mean ± standard deviation, and the statistical significance was expressed at *p* < 0.05. The statistical significance between the groups was evaluated by one-way ANOVA, and calculated with the SPSS 20.0 statistical software (SPSS Inc., Chicago, IL, United States) by Bonferroni multiple comparison test. Figures were drawn using GraphPad Prism 8.0 (San Diego, United States).

## Results

### Optimization of Sequential Extraction and Purification Process of Pumpkin Polysaccharides

**Figure 1:**
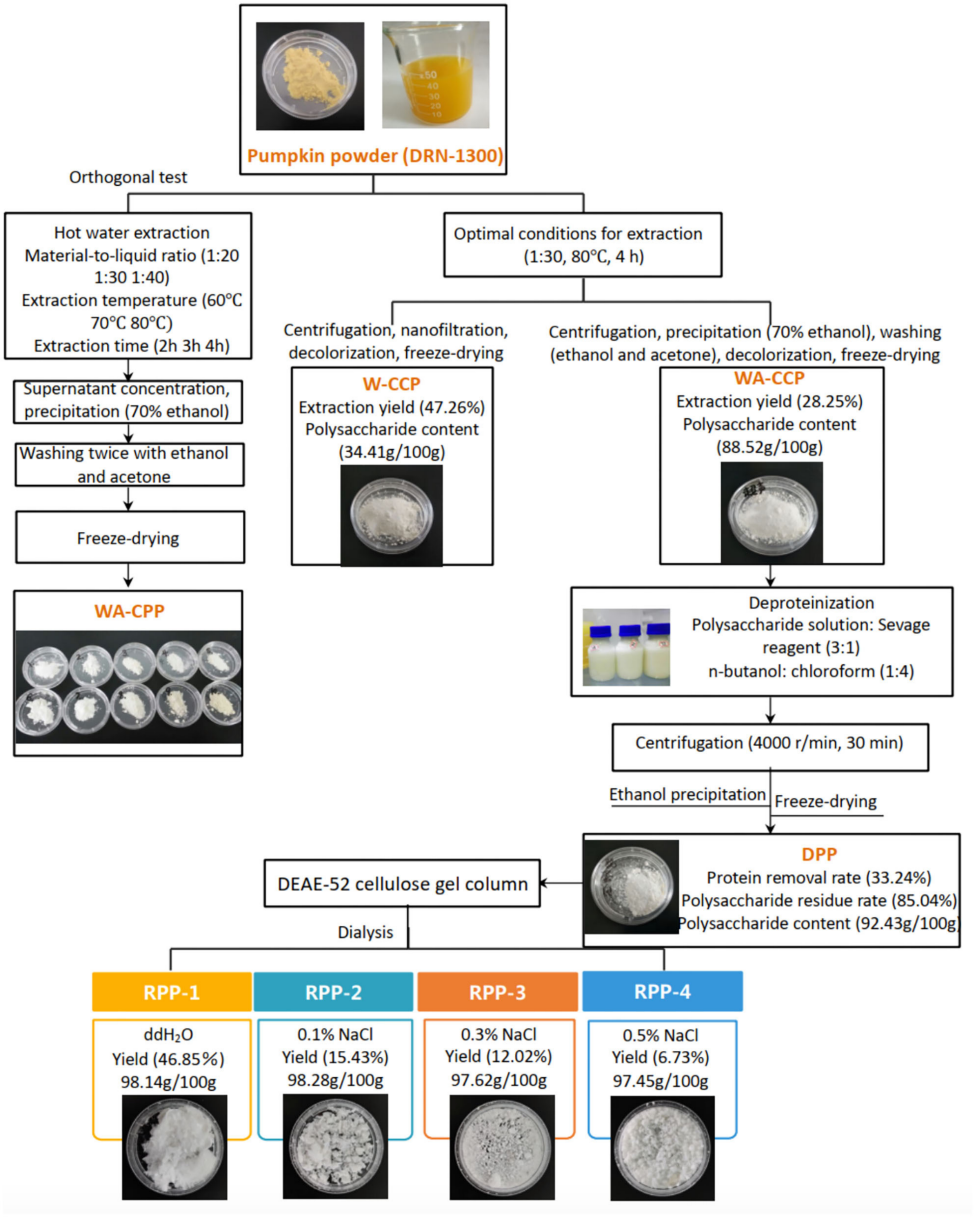
Pumpkin polysaccharide sequential extraction and purification process flow. W-CPP, water-extracted crude pumpkin polysaccharides; WA-CPPs, crude pumpkin polysaccharides extracted by water and alcohol; DPPs, deproteinized pumpkin polysaccharides; RPPs, refined pumpkin polysaccharides.

The hot water extraction method is one of the most common ways to obtain polysaccharides. Comparing the extraction yield, polysaccharide content, and OH radical scavenging ability among 10 extraction conditions, the best extraction condition was that pumpkin powder was added with distilled water with the material-to-liquid ratio of 1:30, extracted at 80°C for 4 h, and then ultrasonicated for 30 min. After centrifugation, concentration, decolorization, and freeze drying, W-CCPs were obtained. WA-CCPs were obtained by ethanol precipitation and organic solvent washing. The extraction yield and polysaccharide content of W-CCPs were 47.26 and 34.41 g/100 g, and WA-CCPs were 28.25 and 88.52 g/100 g, respectively. DPPs were prepared after WA-CCPs were deproteinized, the protein removal rate and the polysaccharide retention rate of DPPs were 33.24 and 85.04%, and the polysaccharide content was 92.43 g/100 g. Then, DPPs were purified with the DEAE-52 cellulose gel column to obtain four refined pumpkin polysaccharide components, RPP-1, RPP-2, RPP-3, and RPP-4. The yields accounted for 46.85, 15.43, 12.02, and 6.73%, respectively. The schematic routes for pumpkin polysaccharide sequential extraction and purification process are graphically depicted in Figure 1. W-CCPs, WA-CCPs, DPPs, and RPPs were analyzed for their physicochemical properties, monosaccharide composition, characterization, and activities.

### Physicochemical Properties of Pumpkin Polysaccharides

**Figure 2:**
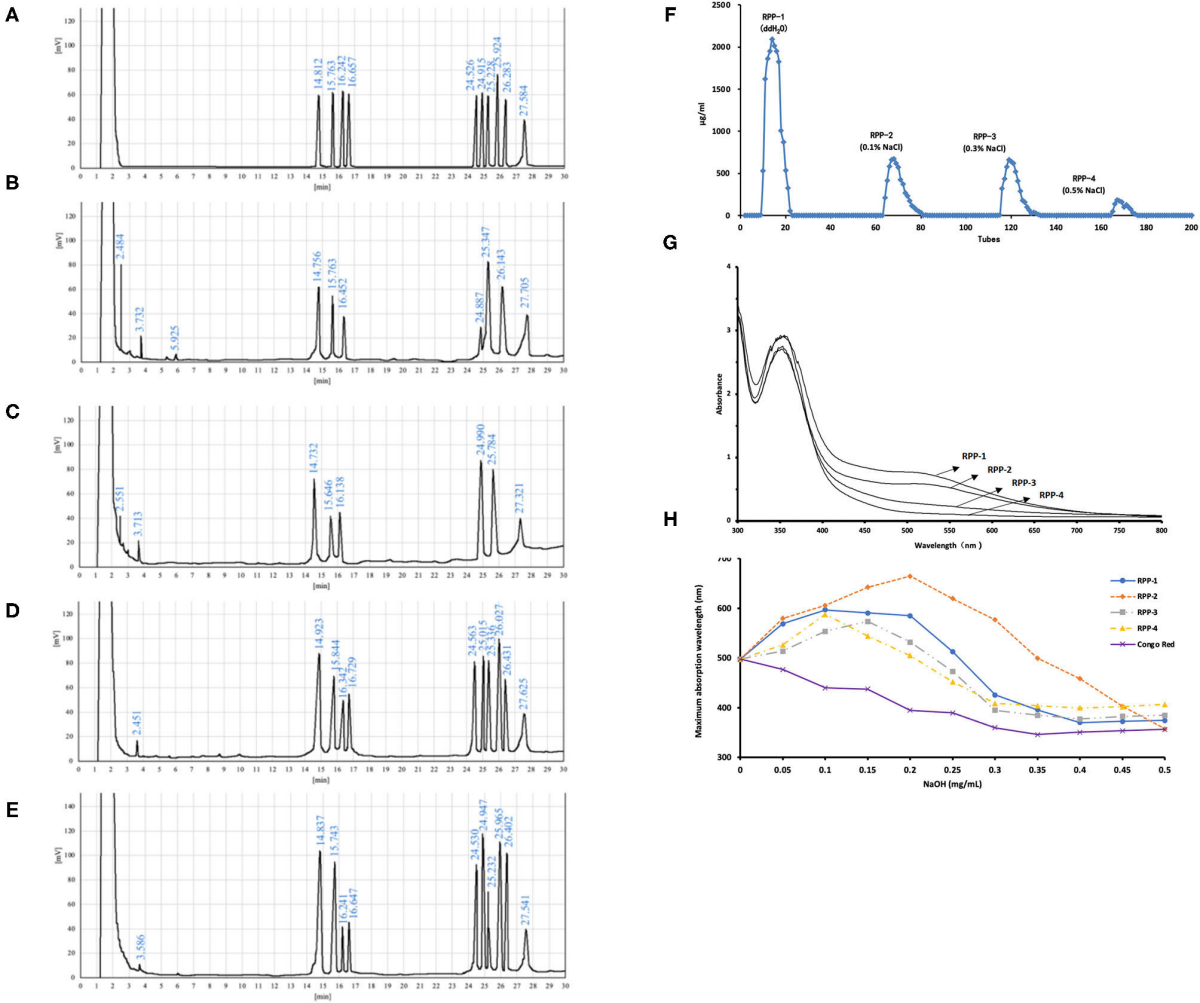
Purification and monosaccharide composition of RPPs and their ultraviolet (UV)-visible (vis) spectra. **(A)** Gas chromatography (GC) of monosaccharides and internal standard, L-rhamnose, D-xylose, D-arabinose, D-ribose, D-fructose, D-mannose, D-glucose, D-galactose, D-fucose, and inositol (from left to right). **(B–E)** Hydrolyzed production of RPP-1, RPP-2, RPP-3, RPP-4, and inositol. Purification of RPPs and their UV-vis spectra. **(F)** DEAE-cellulose anion-exchange chromatography of DPPs. **(G)** UV-vis spectrum of the RPPs and the iodine-potassium iodide reagent. **(H)** Maximum absorption wavelengths of RPPs and Congo red complex increases under alkaline conditions.

The W-CCPs were a light yellow powdered solid, and the WA-CCPs and DPPs were white powdered solid. RPPs were a loosely organized white aggregate, while RPP-4 was slightly flocculent. The samples were all soluble in water, and insoluble in organic solvents such as ethanol, acetone, and chloroform. The physicochemical properties of pumpkin polysaccharides are shown in Table 1. W-CCPs and WA-CCPs were crude polysaccharides with a carbohydrate content of 34.41 and 88.52 g/100 g, DPPs and RPPs contained higher polysaccharide content with 92.43 and over 97 g/100 g. The protein content decreased sequentially after extraction and purification. W-CCPs, WA-CCPs, and DPPs were mixtures that contain reducing sugars, polyphenols, uronic acid, soluble starch, and some amino acids, peptides and other substances. The purified polysaccharide component RPPs did not contain reducing sugars, polyphenols, and soluble starch except RPP-1 and RPP-2 that had a small amount of starch, but all contained uronic acid, amino acids and peptides. DPPs are separated in the DEAE-52 cellulose gel column to obtain RPP-1, RPP-2, RPP-3, and RPP-4, as shown in Figure 2A, of which, RPP-1 is a neutral polysaccharide, and RPP-2, RPP-3, and RPP-4 are all acidic polysaccharides. The components of RPP-1 and RPP-2 were relatively single and did not contain impurities such as protein and nucleic acid, and uronic acid content was 4.32 and 11.18%, respectively. RPP-3 and RPP-4 contained impurities such as protein, and uronic acid content was 19.35 and 23.14%, respectively, which was significantly higher than the corresponding content of RPP-1.

**Table 1:** Physicochemical properties and monosaccharide composition of the pumpkin polysaccharides.

**Sample**	**W-CCP**	**WA-CCP**	**DPP**	**RPP-1**	**RPP-2**	**RPP-3**	**RPP-4**
Extraction yield (%)	47.25 ± 5.23	28.25 ± 4.71	20.34 ± 2.15	Recovery (%) 75.72 ± 4.61			
				46.85 ± 3.24	15.43 ± 2.35	12.02 ± 1.38	6.73 ± 0.46
Carbohydrate (g/100 g)	34.41 ± 2.64	88.52 ± 7.53	92.43 ± 8.41	98.14 ± 3.67	98.28 ± 3.36	97.62 ± 4.17	97.45 ± 3.64
Protein (%)	6.37 ± 0.35	5.45 ± 0.42	3.64 ± 0.13	N.D.	N.D.	0.14 ± 0.03	0.43 ± 0.06
Uronic acid (mg/g)	3.47 ± 0.23	7.27 ± 2.35	7.67 ± 1.46	4.32 ± 0.76	11.18 ± 1.27	19.35 ± 2.47	23.14 ± 2.56
Polyphenol (mg/g)	4.25 ± 0.04	2.48 ± 0.07	1.04 ± 0.01	N.D.	N.D.	N.D.	N.D.
Iodine-potassium iodide reaction (starch)	+	+	+	+	+	N.D.	N.D.
Fehling test (reducing sugars)	+	+	+	N.D.	N.D.	N.D.	N.D.
Ninhydrin reaction (amino acids and peptides)	+	+	+	+	+	+	+
Sugar composition (molar ratio)							
Xyl	-	-	-	1.0	1.0	1.0	1.0
Ara	-	-	-	1.6	1.1	1.7	2.6
Rib	-	-	-	N.D.	N.D.	1.0	1.1
Fru	-	-	-	N.D.	N.D.	1.4	1.4
Man	-	-	-	0.4	3.4	1.3	2.2
Glc	-	-	-	2.7	3.1	1.3	1.1
Gal	-	-	-	2.4	N.D.	2.1	1.8
Rha	-	-	-	2.1	2.9	2.7	3.8
Fuc	-	-	-	N.D.	N.D.	1.2	1.5

*N.D., Not detected; W-CPPs, water-extracted crude pumpkin polysaccharides; WA-CPPs, crude pumpkin polysaccharides extracted by water and alcohol; DPPs, deproteinized pumpkin polysaccharides; RPPs, refined pumpkin polysaccharides. Each value is expressed as the mean ± SD (n = 3)*.

The monosaccharide compositions of the four RPPs are summarized in Table 1 and Figure 2. The peak times of monosaccharide standards in gas chromatogram were: L-rhamnose 14.812 min, D-rrabinose 15.763 min, D-xylose 16.242 min, D-ribose 16.657 min, D-fructose 24.526, D-mannose 24.915 min, D-glucose 25.228 min, D-galactose 25.924 min, L-fucose 27.584 min, and inositol peaked at 27.584 min. The purified pumpkin polysaccharide components RPP-1, RPP-2, RPP-3, and RPP-4 run by GC are shown in Figures 2B–E. RPP-1 and RPP-2 contained Rha, Ara, Xyl, Man, Glc, Gal, and Rha, Ara, Xyl, Man, Glc, separately, while RPP-3 and RPP-4 were all composed of different molar percentages of Rha, Ara, Xyl, Rib, Fru, Man, Glc, Gal, and Fuc. Glc was considered the dominant monosaccharide in RPP-1 and RPP-2, whereas Rha was the predominant monosaccharide in RPP-3 and RPP-4. Moreover, Rib, Fru, and Fuc existed in RPP-3 and RPP-4. According to the comparison between the peak time of the monosaccharide standards and the polysaccharide samples using the area normalization method, through the calculation of the peak area, the molar ratio of the monosaccharide composition of the pumpkin polysaccharides RPP-1, RPP-2, RPP-3, and RPP-4 were obtained and the results are shown in Table 1. The molar ratio of Xyl, Ara, Man, Glc, Gal, and Rha in RPP-1 was 1:1.6:0.4:12.7:2.4:2.1, and Xyl, Ara, Man, Glc, and Rha in RPP-2 was 1:1.1:3.4:13.1:2.9, while nine monosaccharides, Xyl, Ara, Rib, Fru, Man, Glc, Gal, Rha, and Fuc in RPP-3 and RPP-4 were 1:1.7:1:1.4:1.3:1.3:2.1:2.7:1.2 and 1:2.6:1.1:1.4:2.2:0.9:1.8:3.8:1.5, separately.

### Preliminary Structural Characteristics and Microstructures of RPPs

The UV spectrum of RPPs had a characteristic absorption peak of sugars at a wavelength of 220 nm. There were no obvious characteristic absorption peaks between 260 and 280 nm of RPP-1 and RPP-2, indicating that there were no protein and nucleic acid, and that no absorption peak in the visible part showed no pigment after purification. However, RPP-3 and RPP-4 had a slight fluctuation at 260 nm, which was consistent with the protein content data in Table 1. The UV-visible spectrum of the RPPs and the iodine-potassium iodide reagent are shown in Figure 2. After the polysaccharide components RPP-1, RPP-2, RPP-3, and RPP-4 reacted with iodine-potassium iodide, the maximum absorption peak appeared at 350 nm in the UV spectrum. There was no absorption peak over 530 nm, but the intensity of the absorbance of RPPs at this wavelength was weakening, indicating that the spatial conformations of the four components were different. According to literature reports (36), this phenomenon showed that these four polysaccharide components may contain relatively long side chain structures and more branched structures. Congo red is an acid dye that can form complexes with polysaccharides with a triple structure. Compared with Congo red, the maximum absorption wavelength of the complex increases under alkaline conditions. As the concentration of the sodium hydroxide solution gradually increased from 0 to 0.5 mol/L, the maximum absorption wavelengths of the polysaccharide component RPPs all increased first and then decreased, as shown in Figure 2. SEM images of the four polysaccharide components, RPP-1, RPP-2, RPP-3, and RPP-4, are shown in Supplementary Figure 1. The surface morphology of RPP-1 and RPP-2 were irregular fragmented aggregates. Among them, the surface of RPP-1 was relatively flat and smooth with tight combination, while RPP-2 was mainly presented as small clumps, thick, and uneven surface. The surface morphology of RPP-3 was a rough spherical structure, with many fluffy debris. RPP-4 was mainly presented as a wrinkled flocculent structure, loose and porous but not tightly coupled.

**Figure 3:**
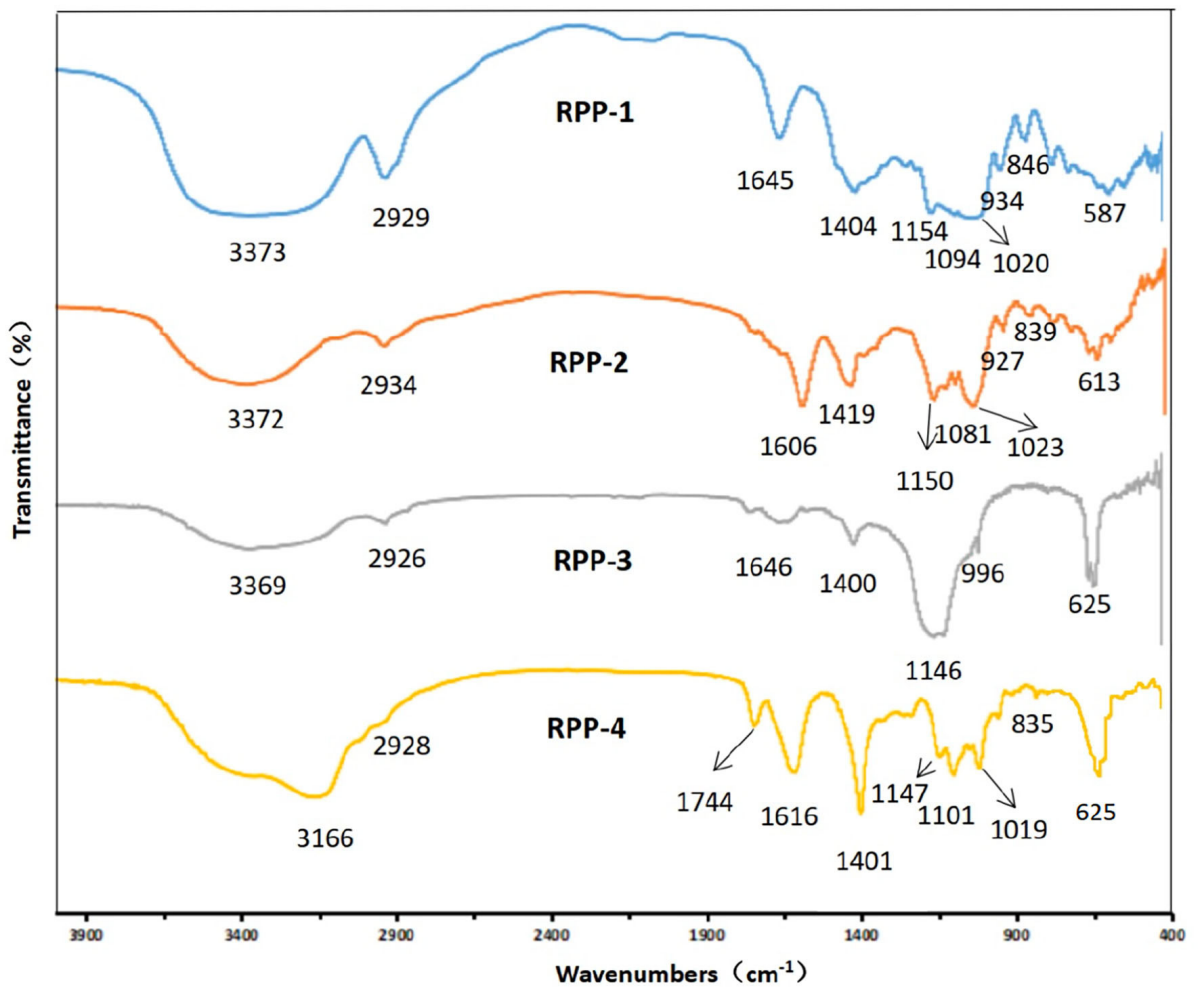
Fourier transform infrared (FT-IR) spectrum of RPPs. RPPs, refined pumpkin polysaccharides. Deproteinized pumpkin polysaccharides were separated in DEAE-52 cellulose gel column to obtain RPP-1, RPP-2, RPP-3, and RPP-4. RPP-1 was a neutral polysaccharide, while RPP-2, RPP-3, and RPP-4 were all acidic polysaccharides.

After chromatography, the infrared scan spectra of RPP-1, RPP-2, RPP-3, and RPP-4 had roughly the same characteristic absorption peak trend, but the peak width and numbers were different, as shown in Figure 3. RPP-1, RPP-2, RPP-3, and RPP-4 had relatively strong wide peaks at 3,373, 3,372, 3,369, and 3,166 cm^−1^, respectively, which is in the range of 3,500–3 200 cm^−*l*^ where O-H stretching vibration peaks. The absorption peaks of RPP-1, RPP-2, and RPP-3 in this range were wider than that of RPP-4, indicating the existence of intermolecular hydrogen bonding (2). RPP-1, RPP-2, RPP-3, and RPP-4 had relatively strong absorption peaks at 2,929, 2,934, 2,926, and 2,928 cm^−1^, respectively, which were C-H stretching vibration peaks, but RPP-4 was relatively weak. Near 1,600 and 1,400 cm^−1^ were derived from the asymmetric and symmetric stretching vibrations of the carboxyl bond (C=O), respectively (37), proving the existence of uronic acid in RPPs and containing -COOH group. In particular, RPP-4 had the largest peak of carboxyl group. The absorption peak of RPP-4 at 1,744 cm^−1^ was the characteristic absorption peak of methoxy groups in pectin polysaccharides, which proves the existence of methoxy groups in RPP-4. RPP-3 had an absorption peak at 1,576 cm^−1^, indicating that there is N-H variable angle vibration. The absorption peak at 1,000–1,200 cm^−1^ is caused by the vibration of the sugar ring backbone C-O and C-C, and has different spectral shapes for polysaccharides composed of different monosaccharides (38). According to Qiao et al. (39), RPP-1, RPP-2, and RPP-4 had three absorption peaks, indicating these three polysaccharides were pyranose, of which near 1,020 cm^−1^ was the C-O stretching vibration absorption peak of the pyranose ring. When pyranose is characterized by absorption around 840 cm^−1^, it generally contains β-type glycosidic bonds, so RPP-1, RPP-2, and RPP-4 were pyranose-containing β-type glycosidic bonds (40). RPP-3 had two absorption peaks at 1,146 and 1,019 cm^−1^, indicating that it had furanose.

### *In vitro* Antioxidant Activities of Four Pumpkin Polysaccharides

**Figure 4:**
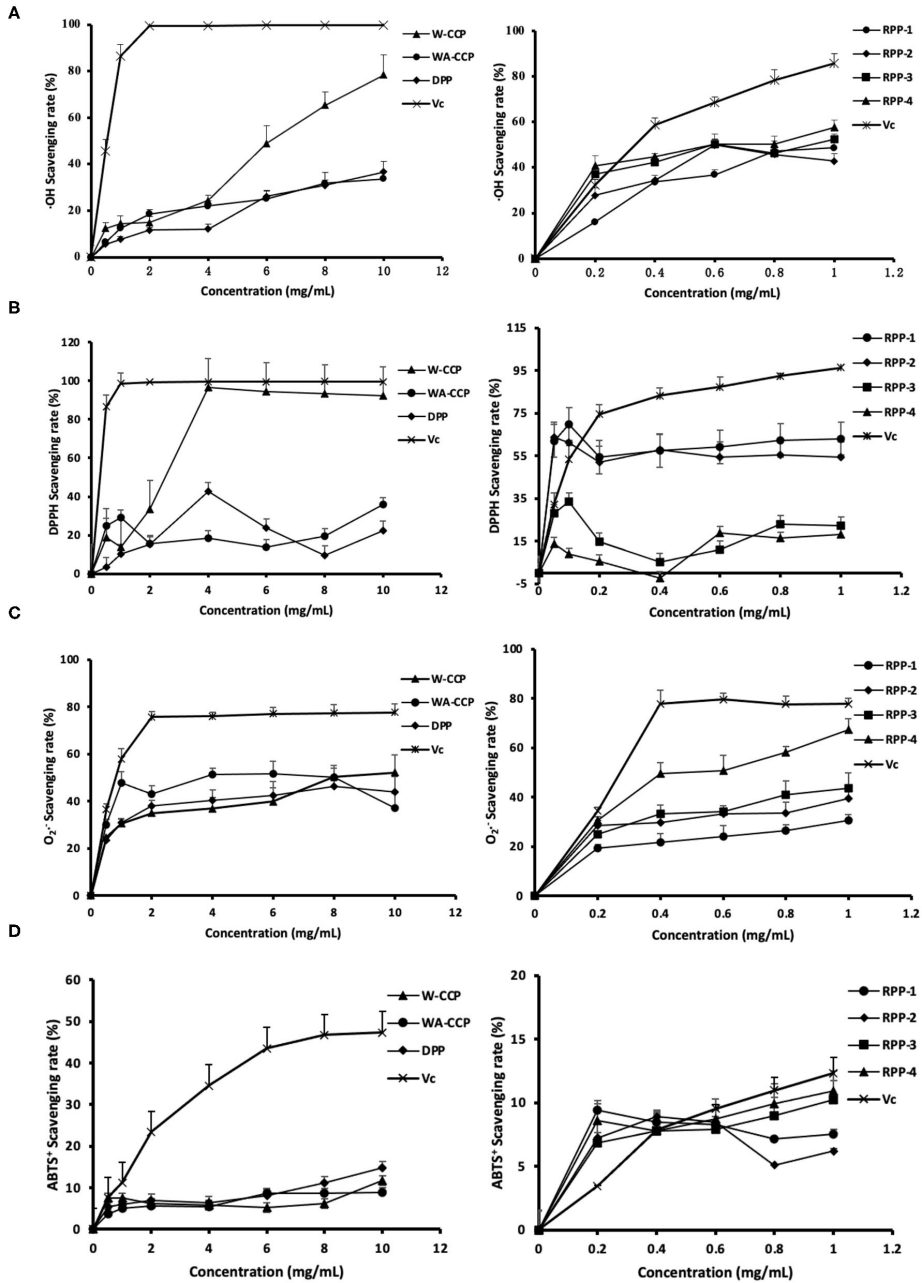
Antioxidant activities of four pumpkin polysaccharides: W-CCPs, WA-CCPs, DPPs, and RPPs. **(A)** Hydroxyl (OH) radical scavenging abilities. **(B)** 1,1-diphenyl-2-picryl-hydrazyl (DPPH) radical scavenging abilities. **(C)** Superoxide anion (${\text{O}}_{2}^{-}$) radical scavenging abilities. **(D)** 2,20-Azino-bis-3-ethylbenzthiazoline- 6-sulphonic acid (ABTS^+^) radical scavenging abilities. V_C_ is the positive control. Each value was represented as mean ± SD of three independent experiments. W-CPPs, water-extracted crude pumpkin polysaccharides; WA-CPPs, crude pumpkin polysaccharides extracted by water and alcohol; DPPs, deproteinized pumpkin polysaccharides; RPPs, refined pumpkin polysaccharides.

The ·OH radical-scavenging abilities of W-CCPs, WA-CCPs, DPPs, RPPs and V_C_ are shown in Figure 4A. Pumpkin polysaccharides subjected to water extraction, alcohol precipitation, deproteinization, and chromatography had certain effects on the scavenging of ·OH radicals. The scavenging rate of ·OH free radicals of the four pumpkin polysaccharides and VC was enhanced with the increase in sample amounts. When W-CCPs were 10 mg/ml, the scavenging ability of ·OH radicals reached 78.36%. The ·OH radical-scavenging abilities of the RPPs after chromatographic purification were similar and higher than that of WA-CCPs and DPPs, indicating that the purification of pumpkin polysaccharides improved its OH radical-scavenging abilities to a certain extent. Moreover, at a concentration of 1 mg/ml, the scavenging rates of the four components of the refined pumpkin polysaccharide were 48.57, 42.83, 52.46, and 57.57%, respectively. Among them, RPP-4 had the highest ability to scavenge OH free radicals. The DPPH radical-scavenging abilities of W-CCPs, WA-CCPs, DPPs, RPPs and V_C_ are shown in Figure 4B. W-CCPs reached 96.69% DPPH radical-scavenging ability at a concentration of 4 mg/ml, which was significantly higher than that of pumpkin polysaccharide after alcohol precipitation and deproteinization. The DPPH radical-scavenging ability curves of the RPPs all showed a trend of first rising and then falling. When the sample concentration reached 1 mg/ml, the DPPH radical-scavenging capabilities of RPP-1, RPP-2, RPP-3, and RPP-4 were 63.13, 54.52, 22.45, and 18.34%, respectively. The clearance effect of RPP-1 and RPP-2 in the chromatographic polysaccharide fraction was better and much higher than that of RPP-3 and RPP-4.

The O_2_·^−^ radical-scavenging abilities of W-CCPs, WA-CCPs, DPPs, RPPs, and VC are shown in Figure 4C. The O_2_·^−^ radicals present in the body come from superoxide anions, which are transformed into H_2_O_2_ and ·OH by the disproportionation reaction, causing damage to the body. The four pumpkin polysaccharides all had the effect of scavenging ${\text{O}}_{2}^{-}$ radicals, as shown in Figure 4C. Before the concentration reaches 8 mg/ml, the effects of the first three had obvious differences, which are in the order of: WA-CCPs > DPPs > W-CCPs, and WA-CCPs could achieve a 50% clearance rate. After chromatography, the removal ability of each component of RPPs was also different in the order of RPP-4, followed by RPP-3 and RPP-2, and the worst was RPP-1. Moreover, at a concentration of 1 mg/ml, the scavenging rates of these four were 67.37, 43.52, 39.37, and 30.61%, respectively. The ABTS^+^ radical-scavenging abilities of W-CCPs, WA-CCPs, DPPs, RPPs, and V_C_ are shown in Figure 4D. ABTS^+^ generates stable free radicals under active oxygen, showing a blue-green color. If it reacts with antioxidant substances, the color will become lighter. The four pumpkin polysaccharides all had a weak ability to remove ABTS^+^, with a removal rate of <20%, and certain concentration dependence. Our results, thus, indicated that the four polysaccharides from pumpkin had positive scavenging capacities on the OH, DPPH, ${\text{O}}_{2}^{-}$, and ABTS^+^ radicals.

### *In vitro α*-Glucosidase and α-Amylase Inhibitory Activities of Four Pumpkin Polysaccharides

**Figure 5:**
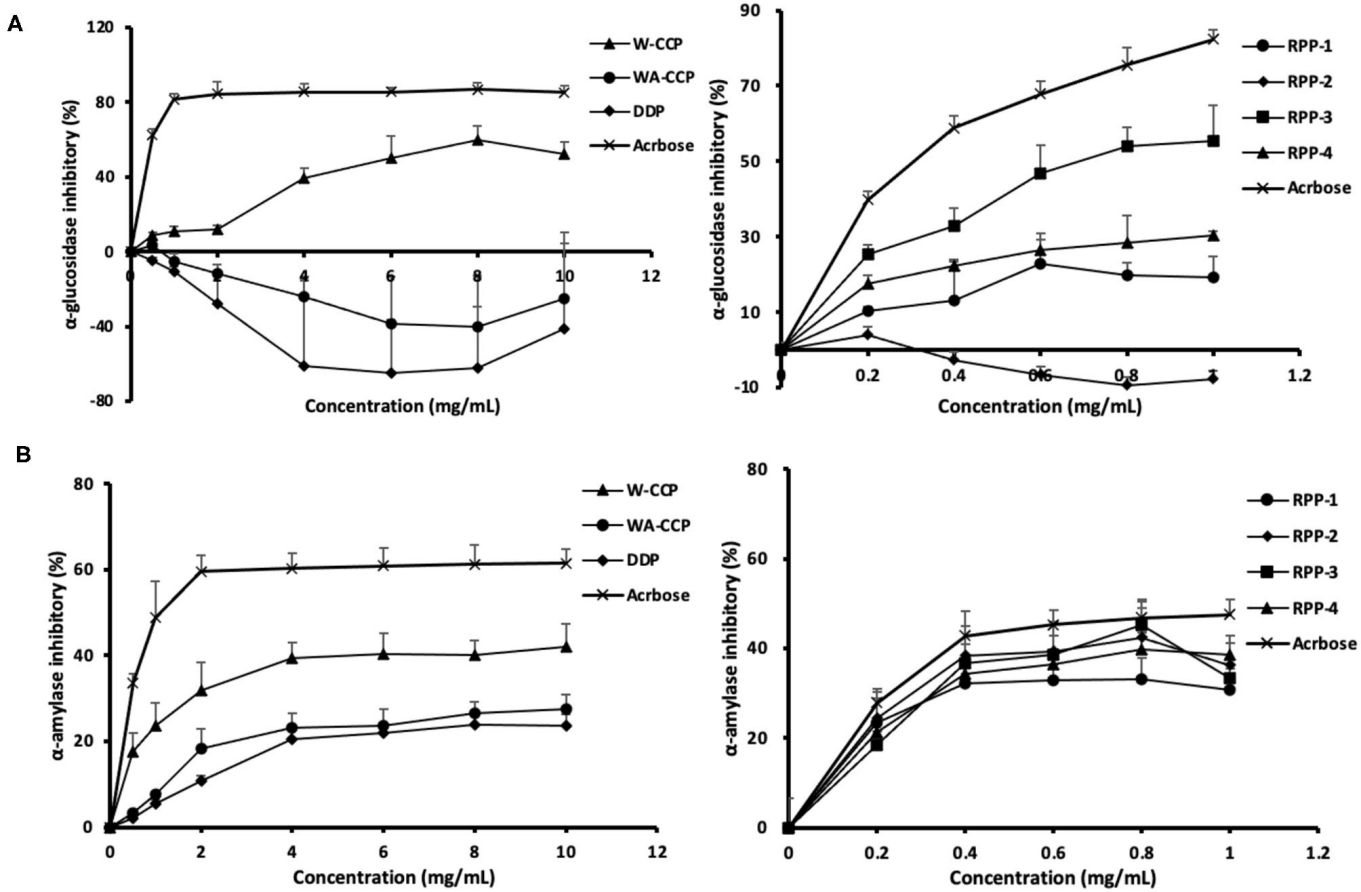
*In vitro* hypoglycemic effects of the four pumpkin polysaccharides: W-CCPs, WA-CCPs, DPPs, and RPPs. **(A)** α-Glucosidase inhibitory activities; **(B)** α-amylase inhibitory activities. Each value was represented as mean ± SD of three independent experiments. W-CPPs, water-extracted crude pumpkin polysaccharides; WA-CPPs, crude pumpkin polysaccharides extracted by water and alcohol; DPPs, deproteinized pumpkin polysaccharides; RPPs, refined pumpkin polysaccharides. Acarbose is the positive control.

The *in vitro* hypoglycemic effects of the four pumpkin polysaccharides were investigated *via* α-glucosidase and α-amylase inhibitory activity assays using acarbose as a positive reference. The α-glucosidase inhibitory activities of W-CCPs, WA-CCPs, DPPs, RPPs, and acarbose are shown in Figure 5A. The W-CCPs had a certain ability to inhibit α-glucosidase, and the inhibition rate was up to 53.9% when the concentration reached 8 mg/ml, while the WA-CCPs and DPPs had no α-glucosidase inhibitory ability. The α-glucosidase inhibitory ability of the purified pumpkin polysaccharides RPP-1, RPP-3, and RPP-4 was enhanced with the increase of concentration, among which RPP-2 even had no inhibitory ability. Moreover, at a concentration of 1 mg/ml, the α-glucosidase inhibitory ability of RPP-3, RPP-4, and RPP-1 reached 55.25, 30.36, and 19.24%, respectively. The α-amylase inhibitory activities of W-CCPs, WA-CCPs, DPPs, RPPs, and acarbose are shown in Figure 5B. α-Amylase can hydrolyze the α-1,4-glycosidic bond inside starch, and the hydrolyzed products are dextrin, oligosaccharides, and monosaccharides. Similarly, the W-CCPs had a higher ability to inhibit α-glucosidase with 42.02 inhibition rate than the WA-CCPs and DPPs, and the inhibition rates of the latter were 27.54 and 23.75%, respectively. The excellent inhibitory effects of W-CCPs against α-amylase and α-glucosidase may be attributed to its simple extraction process and no exposure to organic solvents. This phenomenon was consistent with the results of the antioxidant activities obtained *in vitro* (Figures 4A,B). The purified pumpkin polysaccharide RPPs all exhibited a better inhibitory effect on α-amylase within the range of the determined concentrations. Our results, thus, indicated that the four polysaccharides from pumpkin, especially W-CCPs and RPP-3, had positive α-amylase and α-glucosidase inhibitory capacities.

### *In vitro* Glucose Uptake Inhibitory Activities and Transport Effect of Pumpkin Polysaccharide RPPs

**Figure 6:**
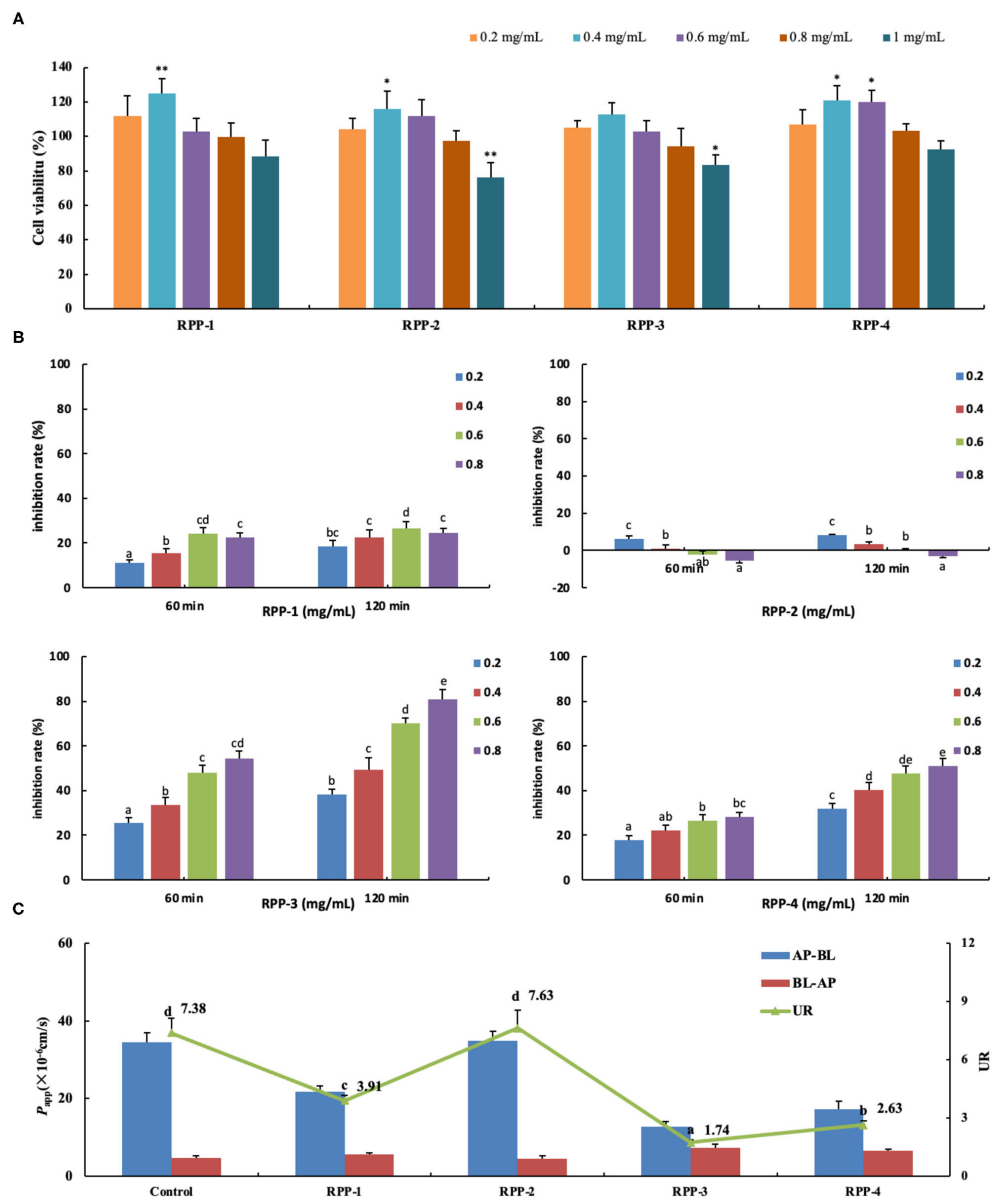
Pumpkin polysaccharide RPPs on the glucose uptake and transport across Caco-2 cell monolayer. **(A)** RPPs on the activity of colon cancer cells Caco-2. **(B)** Inhibition rate of RPPs on Caco-2 glucose uptake. **(C)** RPPs on the transport of glucose across Caco-2 cell monolayer. Each point represents the mean ± SD for at least three independent monolayers (a<b<c<d<e, all *p* < 0.01). RPPs, refined pumpkin polysaccharides; UR, uptake ratio.

When Caco-2 cells were cultured on filter supports for 21 days and differentiated to Caco-2 monolayers, the morphology are shown in Supplementary Figure 2. Under an inverted microscope (Supplementary Figure 2A), the multiple cells were closely connected and arranged in a “paving stone” mosaic arrangement. The well-differentiated Caco-2 cell monolayer contained dense microvilli and neat brush borders (Supplementary Figures 2D,E) and tight junctions among the cells (Supplementary Figure 2F). The formation of tight junctions and microvilli well-mimicked the apical side of the small intestinal epithelial cells facing the intestinal lumen (AP side) and the basolateral membrane (BL side). After the cells were cultured for 21 days, the TEER rose to 499 ± 18 Ω·cm^2^ and remained relatively stable (Figure 6B). Meanwhile, the alkaline phosphatase activity ratio was 16.41 ±0.79 (Table 2) and the lucifer yellow apparent permeability coefficient was 2.36 × 10^−7^ cm/s, which showed that the monolayer was well-differentiated and could be used for a transport experiment (41). The MTT method was used to determine the toxic effects of the four pumpkin polysaccharides, RPP-1, RPP-2, RPP-3, and RPP-4 on Caco-2 cells. In Figure 6A, the results show that the four RPPs have no cytotoxicity at a concentration of 0.2–0.8 mg/ml, and that they could significantly promote cell proliferation at a concentration of 0.4–0.6 mg/ml. Therefore, the sample concentration in the uptake experiment was in the range of 0.2–0.8 mg/ml, and transport experiment was at 0.6 mg/ml.

**Table 2:** Effect of pumpkin polysaccharide RPPs on the transport of glucose across Caco-2 cell monolayer.

	***P***_**app**_ **(×10** ^**−6**^ **cm/s)**		**Uptake Ratio**	**AP-BL permeation (% of control)**	**BL-AP permeation (% of control)**
	**AP-BL**	**BL-AP**			
Control	34.56 ± 2.46^c^	4.68 ± 0.54^a^	7.39 ± 0.77^d^	100^c^	100^a^
RPP-1	21.79 ± 1.58^b^	5.58 ± 0.45^ab^	3.91 ± 0.27^c^	63.05 ± 8.43^b^	119.23 ± 9.36^a^
RPP-2	34.89 ± 2.48^c^	4.57 ± 0.63^a^	7.63 ± 0.94^d^	100.95 ± 9.38^c^	97.65 ± 8.24^a^
RPP-3	12.79 ± 1.28^a^	7.37 ± 0.63^c^	1.74 ± 0.15^a^	37.01 ± 2.59^a^	157.48 ± 14.53^b^
RPP-4	17.21 ± 2.04^ab^	6.54 ± 0.47^bc^	2.63 ± 0.21^b^	69.8 ± 8.0^b^	139.74 ± 12.62^b^

*Each point represents the mean ± SD for at least three independent monolayers (a < b < c < d, all p < 0.01). The uptake ratio (UR) is the ratio of absorption permeability to secretion permeability, which can be expressed as (P_app, AP−BL_/P_app, BL−AP_). AP-BL, apical-to-basolateral; BL-AP, basolateral-to- apical*.

The addition of the four pumpkin polysaccharide RPPs (0, 0.2, 0.4, 0.6, and 0.8 mg/ml) in a 50-mmol/L glucose medium changed the inhibition rate of glucose uptake in the Caco-2 monolayer after the 21-day culture (Figure 6B). Under the same sample concentration and reaction time, RPP-3 had the best inhibitory effect (80.8%, 120 min) on glucose uptake in the Caco-2 monolayer, followed by RPP-4 and RPP-1. With the increase of sample concentration and reaction time, the inhibitory effect of these three RPPs showed a trend of increasing, but RPP-1 showed a slight drop at 0.8 mg/ml concentration. Moreover, RPP-2 had a slight inhibitory effect of glucose uptake in the Caco-2 monolayer under 0.2 mg/ml concentration, while the inhibitory effect of RPP-2 was reduced and even had a promoting effect of glucose uptake. The effect of RPPs on the transport of glucose across the Caco-2 cell monolayer is shown in Figure 6C, and different uptake ratios (URs) of glucose transport in the presence of pumpkin polysaccharide RPPs are shown in Table 3. The addition of RPP-3, RPP-4, and RPP-1 significantly decreased the rate of glucose transport across the cell monolayer to 1.74, 2.63, and 3.91, respectively (*p* < 0.01). The glucose permeation level of RPP-3 in the AP-BL direction was lowest at 12.79 ± 1.28, while in the BL-AP direction it was highest at 7.37 ± 0.63 among the other groups (*p* < 0.01). It indicated that RPP-3 could inhibit the absorption and promote the excretion of glucose in Caco-2 cells. In addition, RPP-2 did not change the transport of glucose across the cell monolayer.

**Table 3:** Relationship between alkaline phosphatase activity and culture time of Caco-2 monolayer.

**t/d**	**Ratio of alkaline phosphatase**	**Activity (U/g protein)**
5	1.63 ± 0.09^a^	22.36 ± 2.32^a^
9	2.98 ± 0.25^a^	21.38 ± 2.64^a^
13	6.73 ± 0.58^b^	21.79 ± 2.83^a^
17	14.27 ± 1.35^c^	62.34 ± 5.58^b^
21	16.41 ± 0.79^c^	68.92 ± 6.47^b^

*Alkaline phosphatase is a marker enzyme of the brush border differentiated during the formation of Caco-2 cell monolayer. Alkaline phosphatase activity can mark the degree of differentiation of cells to a certain extent. With the increase in culture time, alkaline phosphatase activity in the cells increases, and the cells form polar differentiation. Each value was represented as mean ± SD of three independent experiments (a < b < c < d, all p < 0.01)*.

### Pumpkin Polysaccharide Treatment Alleviates Blood Glucose and Lipid Metabolic Disturbance in T2DM Mice

During the experiment, the bodyweight of the mice in the normal diet group increased rapidly for 1–5 weeks and remained stable for 6–10 weeks. The weight of the mice in the HFD of the pumpkin polysaccharide intervention group increased rapidly for 1–5 weeks, and was significantly higher than that of the mice in the normal diet group. In the 5th week, the weight of the mice was significantly reduced after STZ injection. The model mice had typical symptoms of polydipsia, polyphagia, and polyuria, and their body weight was significantly lower than that of the normal diet group after 1 week. After the diabetes model was successfully established on the 6th week, the mice in the HFD group received drug intervention. One week after intervention with pumpkin polysaccharide (the 8 week), the weight of the mice in the HFD group slowly recovered, and the final weight was 22.57 ± 1.26 g, which was significantly lower than that of the normal group, which was 25.37 ± 2.26 g. Through the administration period, the weight of the mice in the model group presented a downward trend, from 23.38 to 21.55 g. The bodyweight of the mice in the PPS.L and PPS.H groups remained almost stable, and slightly higher than during the observation period.

**Figure 7:**
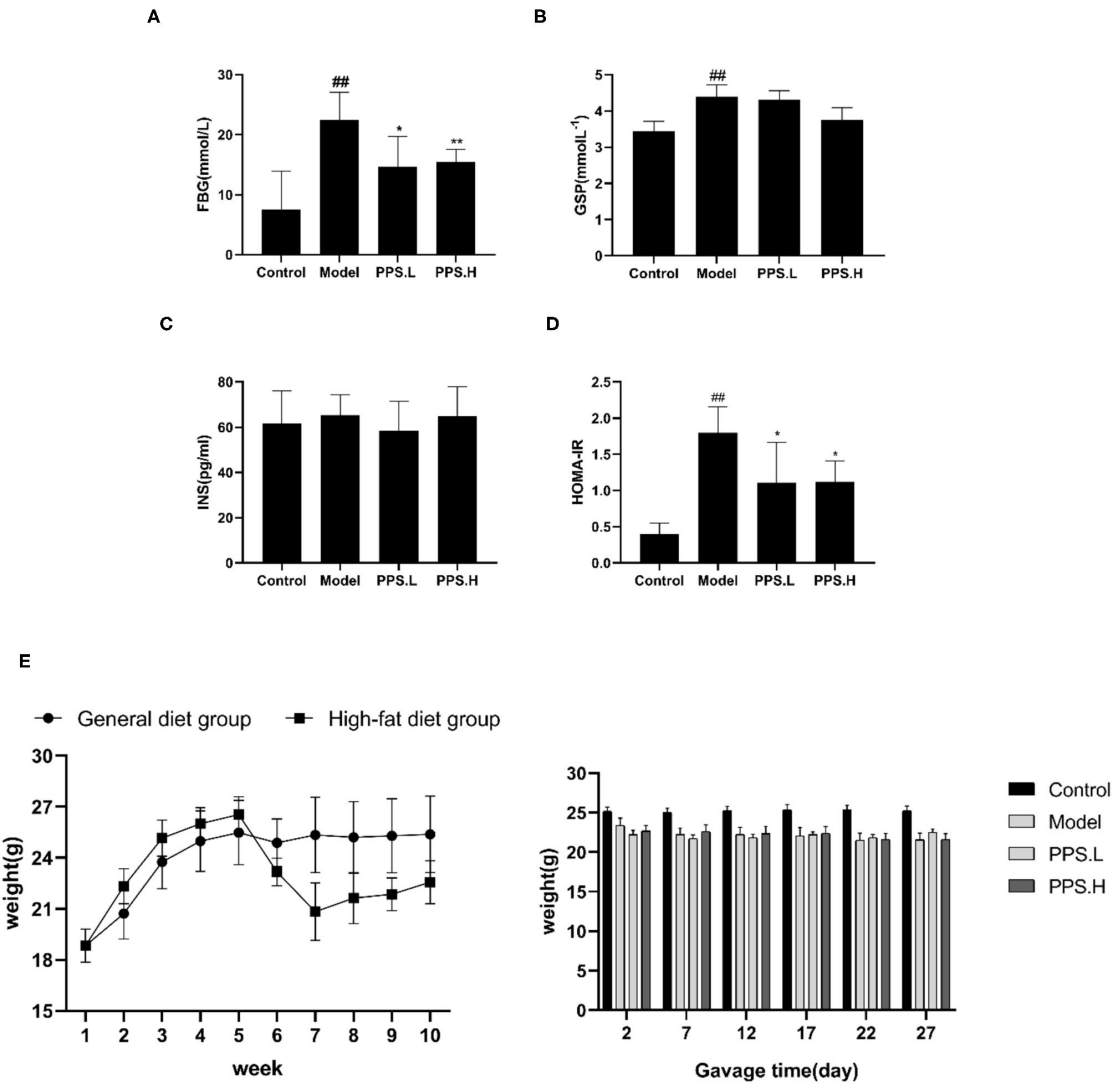
Effect of crude pumpkin polysaccharide on plasma sugar related indexes and weight in mice for 4 weeks. **(A)** Fasting blood glucose (FBG). **(B)** Glycosylatedplasma protein (GSP). **(C)** Insulin (INS). **(D)** Homeostasis model assessment-insulin resistance index (HOMA-IR). **(E)** Body weight of mice. Eight mice in each group. Values are expressed as mean ± SD. ^##^*p* < 0.01, ^#^*p* < 0.05 vs. control group; **p < 0.01, *p < 0.05 vs. model group. PPS.L, pumpkin crude polysaccharide low-dose group (diabetic rats, gavage with W-CCPs at 600 mg/kg b.w.); PPS.H, pumpkin crude polysaccharide high-dose group (diabetic rats, gavage with W-CCPs at 1,200 mg/kg b.w.).

**Figure 8:**
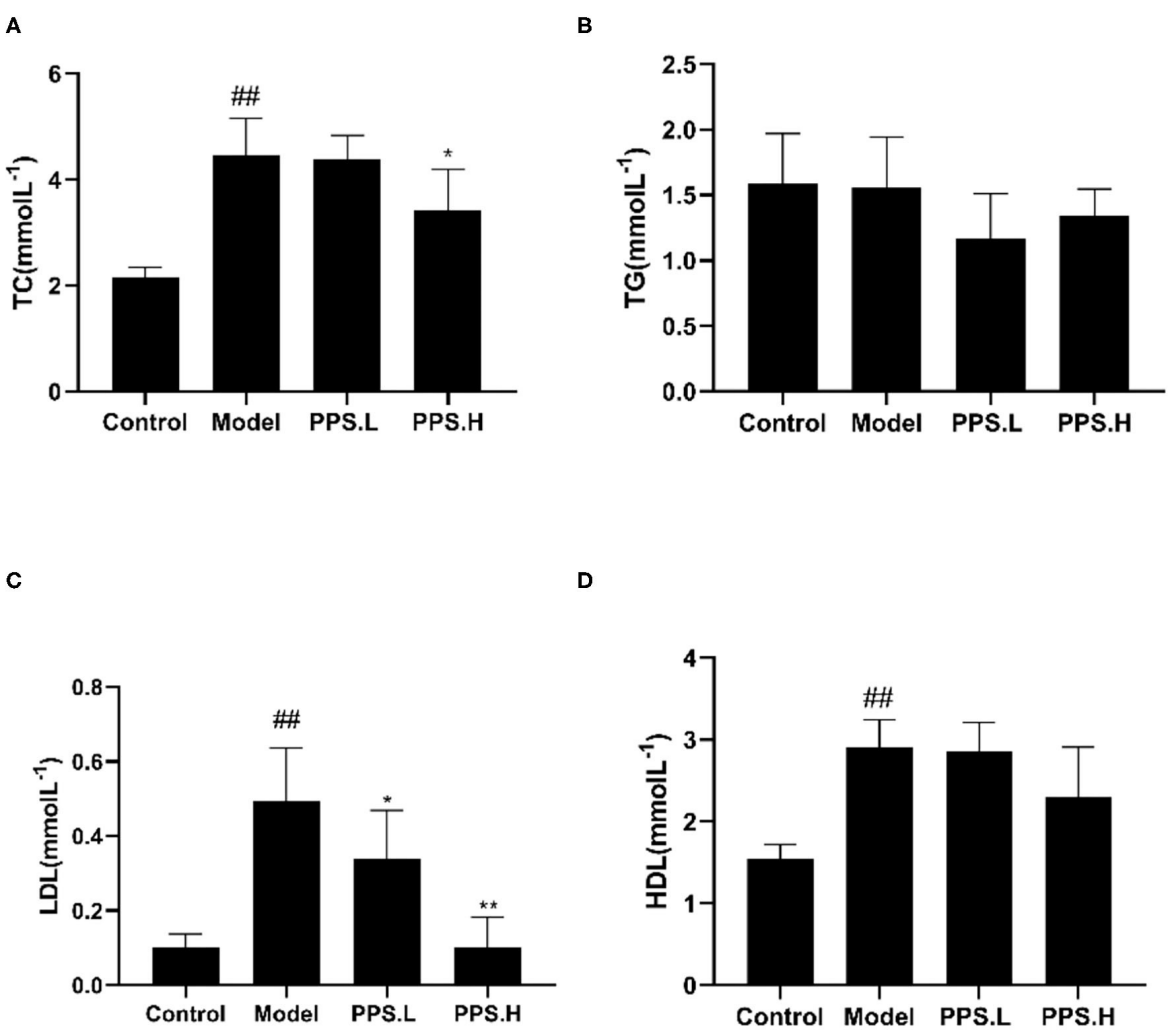
Effect of crude pumpkin polysaccharide on the plasma lipids in mice. **(A)** Total cholesterol (TC). **(B)** Triglycerides (TG). **(C)** Low-density lipoprotein (LDL). **(D)** High density lipoprotein (HDL). Eight mice in each group. Values are expressed as mean ± SD. ^##^*p* < 0.01, ^#^*p* < 0.05 vs. control group; **p < 0.01, *p < 0.05 vs. model group. PPS.L, pumpkin crude polysaccharide low-dose group (diabetic rats, gavage with W-CCPs at 600 mg/kg b.w.); PPS.H, pumpkin crude polysaccharide high-dose group (diabetic rats, gavage with W-CCPs at 1,200 mg/kg b.w.).

The FBG of the normal control mice remained below 7 mmol/L, while the blood glucose of the model group mice was always at a high level and showed an upward trend with the highest value at 23.05 ± 5.03 mmol/L after 4 weeks. After 4 weeks of administration, the PPS.L and PPS.H groups could significantly lower the FBG of mice by 34.06 and 37.87%, respectively (*p* < 0.05). The FBG and GSP of the model group was significantly higher than those of the control group (*p* < 0.01). Compared with the model, the GSP and INS of each drug intervention group decreased, but there was no significant difference observed. Among them, the FBG and HOMA-IR of the PPS.H group were remarkably lower than those of the model group (*p* < 0.01), and the symptoms of insulin resistance were, thus, improved (Figures 7A–E). The above results indicated that pumpkin polysaccharides could reduce the levels of FBG and GSP, and improve the insulin resistance of mice. Compared with the normal group, the plasma TC, HDL, and LDL levels of the model group were significantly higher (*p* < 0.01), pointing out that the mice in the model group had dyslipidemia. After 4 weeks of pumpkin polysaccharide treatment, the plasma TC and LDL levels of the PPS.H group were significantly lower than those of the model group, and the serum HDL and TG level showed a decreasing trend (Figures 8A–D).

### Pumpkin Polysaccharide Treatment Modulated Gut Microbiota Structure in T2DM Mice

**Figure 9:**
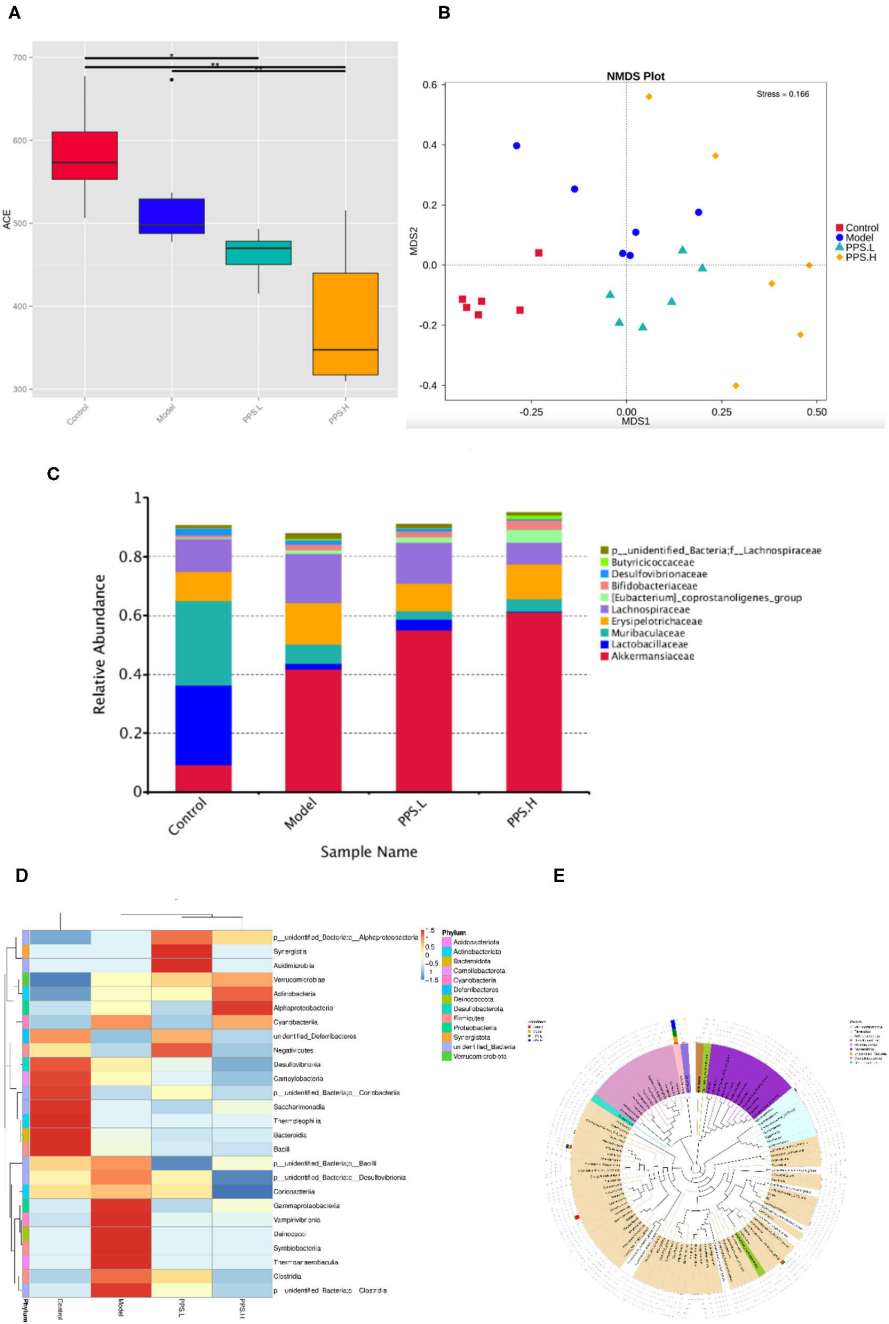
Regulation of PPS on the imbalance of intestinal microbiota in T2DM mice. All mice were sacrificed and colonic samples were collected for 16SrDNA gene sequencing. **(A)** Alpha diversity index-observed species. **(B)** Beta-diversity-NMDS. **(C)** Alteration of gut microbiota composition at family level. **(D)** Heatmap of genera level of each sample. **(E)** Phylogenetic tree of the genus level.

Many studies have reported that gut microbial metabolism disorders may be an important cause of metabolic disorders. In order to evaluate the effect of pumpkin polysaccharides on the gut microbiota of T2DM mice, barcode pyrosequencing technology was used to analyze the structural changes in the gut microbiota of mice in the four groups. The alpha diversity and beta diversity were calculated with the QIIME2 software. Alpha diversity refers to the diversity in a specific area or ecosystem. Through the Alpha diversity index analysis of the differences between groups, it can be judged whether there are significant differences between each group (42) or observed species (Figure 9A) illustrates that the four groups had significant differences (*p* < 0.05). Next, the β diversity of the four groups was analyzed by Non-Metric Dimensional Score (NMDS) to show that the clusters of the gut microbiota in each group were roughly separated. that only the model group was distributed in the first and second quadrants, and that PPS.H and PPS.L were closer to the control group (Figure 9B). The above results indicated that the crude pumpkin polysaccharide treatment significantly changed the intestinal microbial community of mice. To investigate the changes in specific bacteria, an OTU table was generated through analysis, which can reflect the classification of bacteria and quantify relative bacterial abundance. Comparing with the database Silva138 for species annotation statistics (43), it was found that there are a total of 1,210 OTUs, of which the number of OTUs that can be annotated to the database was 1,204 (99.5%). As compared with the control group, the abundances of *Verrucomicrobiota, Proteobacteria*, and *Actinobacteria* were increased, and those of *Firmicutes* and *Bacteroidetes* were decreased at the phylum level in the model and PPS groups. At the family level, the abundances of *Erysipelotrichaceae* and *Lactobacillaceae* were decreased in both the PPS.L and PPS.H groups compared with the model group. Overall, the crude pumpkin polysaccharide treatment increased the relative abundance of the intestinal flora of T2DM mice (Figure 9C). Based on the heat map analysis, we next determined specific genus differences between the four groups. As revealed in Figures 9D,E, the abundance of 21 genera indicates the difference in the gut microbial community of mice with different treatments, which suggests that the remission of T2DM by PPS may be connected to a subset of the bacterial taxa. In the model group, higher abundances of *Clostridia, Thermoanaerobaculia, Symbiobacteriia, Deinococci, Vampirivibrionia, Gammaproteobacterial*, and *Corio bacteria*, accompanied by low levels of *Negativicutes* and *Saccharimonadia*, were observed,. Nevertheless, the PPS treatment reversed the above changes.

**Figure 10:**
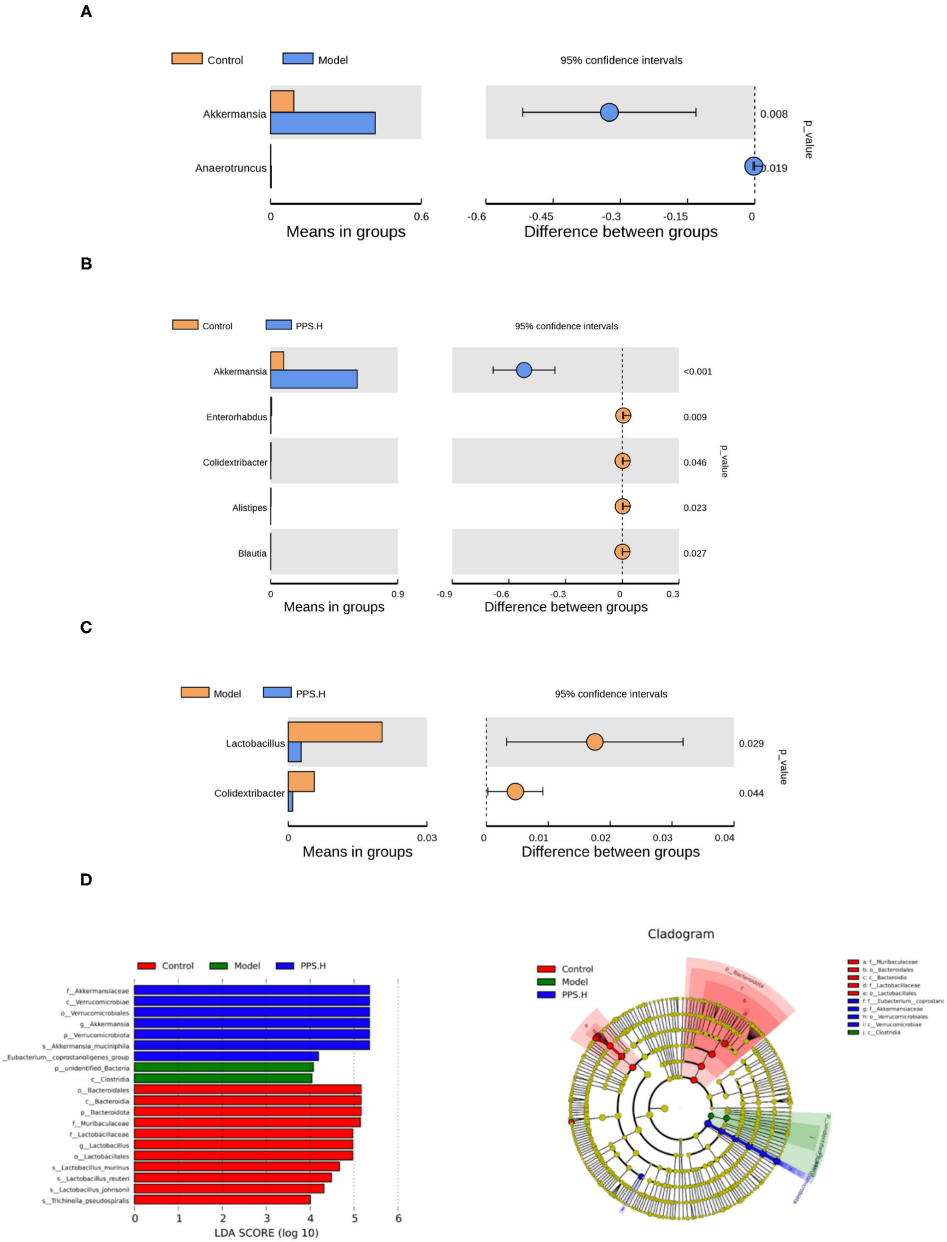
Species differences between *t*-test groups of control and model. **(A)** Control and PPS.H. **(B)** Model and PPS.H. **(C)** At genus level. **(D)** Linear discriminant analysis (LDA) and LDA of effect size (LEFSe) of different groups of microbial flora. The maximum difference between groups after LDA uses a threshold score > 4.

Further starting from the abundance of species at different levels, different species could be obtained through conventional *T*-tests. At the genus level, compared with the normal group, the abundance of *Akkermansia* and *Anaerotruncus* increased significantly in the model group (*p* < 0.05) (Figure 10A). In the PPS.H group, the abundance of *Akkermania* also increased significantly (*p* < 0.05), accompanied by remarkably low levels of *Enterorhabdus, Colidextribacter, Alistipes*, and *Blauti* (Figure 10B). Compared with the model group, the abundance of *Lactobacillus* and *Colidextribacter* decreased significantly in the PPS.H group (*p* < 0.05) (Figure 10C). Finally, the linear discriminant analysis effect size (LEFSE) method was used to compare the composition of the gut microbiota of the three test groups. There were significant differences in the structural composition of the gut microbiota among them (Figure 10D). A total of 20 biomarkers were detected at the class level; *Bacteroidia, Clostridia*, and *Verrucomicrobiae* were biomarkers of the control, model, and PPS groups, respectively. At the family level, *Lactobacillaceae* and *Muribaculaceae* were the biomarkers of the control group, and *Akkermansiaceae* was the biomarker of the PPS.H group (Figure 10D).

## Discussion

Four pumpkin polysaccharide samples, W-CCPs, WA-CCPs, DPPs, and RPPs, were sequentially extracted and purified by hot water extraction, water extraction and alcohol precipitation, deproteinization, and DEAE-52 cellulose gel column. W-CCPs, WA-CCPs, and DPPs are mixtures that contain reducing sugars, polyphenols, uronic acid, and soluble starch, and some amino acids, peptides, and other substances, while the purity of RPPs exceeds 97%, and they do not contain reducing sugars and polyphenols. The protein content decreased, and polysaccharide content increased sequentially after extraction and purification. It shows that the decolorization, deproteinization, and cellulose gel column processes play a certain role in purification. RPP-1 is a neutral polysaccharide, while RPP-2, RPP-3, and RPP-4 are all acidic polysaccharides. This is similar to Zhu et al. (5) and ShanChen et al. (21), who used cellulose DEAE-52 to separate pumpkin polysaccharides and obtain two acidic polysaccharides, but the solubility of the elution solution is not exactly the same. RPP-3 and RPP-4 contain higher protein and uronic acid content than RPP-1 and RPP-2. The content of uronic acid and sulfuric acid groups in the polysaccharide component may be related to their antioxidant potentials (44). Some polysaccharides (glycoproteins) linked to proteins might have stronger biological activity than those without protein, but that requires further verification and discussion by experiment.

Refined pumpkin polysaccharides may contain relatively long side chain, branched structures, and triple helical structure. According to literature reports (36), the maximum absorption peak appeared at 350 nm of RPPs reacting with iodine-potassium iodide shows that these four polysaccharide components may contain relatively long side chain structures and more branched structures. The combination of Congo red and polysaccharide components also confirms that RPPs all have a triple helical structure. This was due to the combination of Congo red and the polysaccharide component with a triple structure, which formed a special complex under weakly alkaline conditions and led to an increase in the maximum absorption wavelength, as shown in Figure 1. The alkalinity continued to increase, then the triple helix was unraveled, and the complex cannot be formed, so the maximum absorption wavelength of the reaction system decreased. The infrared spectrum results show that RPPs contained -COOH group (37, 45); especially, RPP-4 has a large peak of carboxyl and methoxy groups. This is consistent with the higher uronic acid level in RPP-4, as shown in Table 1. The porosity of PPRs range from compact to lose, and the shape ranged from flaky to spherical. This may be related to the extraction method, chemical properties, and structural characterization of pumpkin polysaccharides. The results of monosaccharide compositions of the four RPPs are different from those of previous reports. Zhu et al. demonstrated that a polysaccharide from pumpkin fruit are mainly composed of Ara, Man, Glc, and Gal, with a molar ratio of 1:7.79:70.32:7.05 (5). ShanChen et al. demonstrated that two purified polysaccharides, which were obtained from pumpkin with the combination of an aqueous two-phase system and DEAE cellulose-52 column chromatography, are mainly composed of Glu, Gal, and Ara, with a molar ratio of 14.5:1.6:1.2:1 (21). Wang et al. obtained a polysaccharide from pumpkin seeds, which is composed of Man, Glc, and Gal, with a molar ratio of 1:4.26:5.78, by ultrasound-assisted extraction and two-step column chromatography (46). These show that different extraction methods have an impact on monosaccharide types and molar ratios of the pumpkin polysaccharide obtained.

Carbohydrates are enzymatically decomposed by α-glucosidase, leading to increased blood sugar. During removal, the sample inhibits the activity of α-glucosidase, which shows the decomposition of carbohydrates and reduces the absorbance. In this study, the pumpkin polysaccharides exhibit excellent radical-scavenging ability assay, α-glucosidase and α-amylase inhibition assay, especially W-CCPs, RPP-3, and RPP-4, which are positively correlated with the concentration of the solution. In general, W-CCPs, RPP-3, and RPP-4, which have stronger antioxidant activity, also have better α-glucosidase and α-amylase inhibitory activities. According to previous reports, similar results have been observed that extracted or molecularly modified pumpkin polysaccharides have better *in vitro* antioxidant and hypoglycemic activities (9–12). The non-purified pumpkin polysaccharides (WA-CCPs, DPPs), which were extracted by hot water extraction and water extraction and alcohol precipitation, have lower *in vitro* antioxidant and hypoglycemic activities than the hot water-extracted pumpkin polysaccharide, W-CCPs. This result shows that although sequential extraction improved polysaccharide yield, the antioxidant and hypoglycemic effects did decrease. It may be because the biological activity of pumpkin polysaccharide was destroyed by the increase in extraction steps, time, and the application of organic reagents. Among the RPPs, RPP-3 and RPP-4 had better OH and ${\text{O}}_{2}^{-}$ radical-scavenging abilities, as shown in Figure 4, which indicates that the higher uronic acid content and sulfuric acid groups in the composition of pumpkin polysaccharides, the stronger their antioxidant capacities are (44). Then, RPP-3 and RPP-4 had a significant inhibitory effect on glucose absorption in the Caco-2 monolayer, while RPP-2 had no inhibitory effect. RPP-3 could not only inhibit the uptake of glucose in the Caco-2 cells, but it promoted the excretion of glucose, which can be developed as an additive component of natural medicine or functional food to control blood sugar. These confirm the α-glucosidase and α-amylase inhibition assay results and show the excellent *in vitro* hypoglycemic activity. By comprehensive comparison, the water extraction method used to obtain pumpkin polysaccharides has been found to be relatively simple, highly efficient, and has high biological activity, so it is most suitable for extracting pumpkin polysaccharides for future animal experiments and intervention trials.

Simultaneously, the W-CCPs show an outstanding·OH, DPPH, and O_2_·^−^ scavenging ability in all the samples, as well as α-glucosidase, α-amylase inhibitory activity, so they were selected as an intervention drug for animal experiments. In the *in vivo* experiment, the pumpkin polysaccharide treatment (PPS.H group, namely 1,200 mg/kg W-CCPs) significantly improved the symptoms of diabetic mice by lowering FBG and reducing insulin resistance, which is consistent with previous information that pumpkin or pumpkin polysaccharides had anti-hyperglycemic, anti-hyperlipidemia, and antioxidant effects (4, 8, 47, 48). Our studies show that the hypoglycemic effect of pumpkin polysaccharides can be partially explained by the decrease in glucose absorption rate, increase in peripheral glucose utilization, and removal of free radicals. T2DM can not only cause the disorder of glucose metabolism in the body, but it also has a great destructive effect on the lipid metabolism of the body, and it eventually leads to abnormalities in various indexes of blood lipids in the body (49). In our study, the crude pumpkin polysaccharide treatment significantly lowered the plasma TC, TG, and LDL levels, which is consistent with previous studies on pumpkin polysaccharides (8), acid hydrolysates of pumpkin polysaccharides (4), and pumpkin crude extract (50). Interestingly, in our experiment, the HDL level of mice in the model group was highest after 4 weeks of treatment, which is contrary to the results of other studies. The research of Lu shows that the blood sugar reduction of pumpkin acid hydrolyzed polysaccharide is very complicated and includes a series of complex cell signal transduction and related genes and proteins, and that the treatment is slow (4). However, the LDL/HDL values of the four groups of the control, model, pumpkin polysaccharide low-dose, and high-dose groups are calculated to be 0.13, 0.169, 0.1184, and 0.044, respectively. The trend of these ratios was in line with the trend of dyslipidemia in diabetic mice. The HDL value of the model group increased, and the increase is a compensatory behavior of the body after LDL increased significantly. Therefore, for studies on the use of food-derived active substances to treat diabetes, it is recommended to appropriately extend the time of administration, and 6–8 weeks is recommended.

In addition, studies have reported that active ingredients in many food sources have a good intervention effect on T2DM, especially polysaccharides from mushrooms (cordyceps sinensis, omphalialapidescens, richolomamongolicum, aroniamelanocarpa, red ginseng, and shiitake), vegetables and fruits (red pepper, soybeans, purple carrots, potatoes, momordicacharantia, cucumber, eggplant, celery), and cereals (wheat, rice, corn, oat, barley, sorghum) (6). Pumpkin polysaccharides are also considered to be effective regulators of gut microbiota, which can nourish certain useful microorganisms to play an active role in controlling metabolic diseases such as diabetes (7). In our study, by 16S rDNA analysis, the supplementation of pumpkin polysaccharides changed the abundance of the gut microbiota of mice and reshaped the composition. At the phylum level, *Verrucobacteria, Proteobacteria*, and *Actinomycetes* increased, and *Firmicutes* and *Bacteroides* decreased in the model group, which is consistent with previous studies on flora disorders of T2DM (18, 51, 52). A total of 20 biomarkers are detected, and at the class level, *Bacteroidia, Clostridia*, and *Verrucomicrobiae* are biomarkers in the control, model, and PPS groups, respectively. *Clostridia* contains a lot of opportunistic pathogens, such as *Clostridium clostridioforme, Clostridium hathewayi, Clostridium symbiosum*, and *Eggerthella sp*., which have been confirmed as biomarkers in the microbiota of patients with T2DM (52). When MyD88-deficient non-obese diabetic mice (NOD) fecal microorganisms were administered to NOD mice, there is a promotion in *Clostridiaceae* as well as *Lachnospiraceae* plus a downfall in *Lactobacillaceae* (53). These demonstrate that the transfer of gut microbiota from diabetes-protected MyD88-deficient NOD mice can reduce insulitis and significantly delay the onset of diabetes. Simultaneously, at the family level, *Akkermansiaceae* is the biomarker of the PPS.H group. The *Verrucomicrobia* flora is enriched in the mucus layer of the intestine, and its representative bacteria is *Akkermansia muciniphila* (AKK bacteria). The research by Shin et al. showed that the abundance of AKK bacteria in the intestine of T2DM mice treated with metformin was significantly increased (54). In *in vitro* experiments, the relative abundance of AKK bacteria also increased when metformin was added to the culture medium for fecal bacteria (55). AKK bacteria may be a potential probiotic for the treatment of diabetes, and they are also the only intestinal microbes that can be cultured at present. At the same time, they have become a key organism at the mucosal interface between the intestinal lumen and host cells because of their special performance in the degradation process of mucin. This is related to inflammatory bowel disease, obesity, colitis, type 1/2 diabetes, and other metabolic disorders (56). A clinical study has found that pasteurized AKK improves insulin sensitivity (28.62 ± 7.02%), insulinemia (−34.08 ± 7.12%), and plasma TC in overweight/obese insulin-resistant volunteers (57). These confirm that pumpkin polysaccharides could promote the proliferation of *Akkermansia* to recover from T2DM, which is related to the release of various by-products, such as acetic acid, when AKK degrades mucin (58).

The *t*-test results show that both PPS.L and PPS.H decreased *Erysipelotrichaceae*, which has been confirmed to be extremely sensitive to changes in the concentration of phenyl sulfate and has a strong correlation with diabetic nephropathy. The intestinal microbial-derived metabolite phenol undergoes a sulfation reaction in the liver to synthesize phenyl sulfate. After phenyl sulfate enters the kidney through the blood, it will cause damage and loss of kidney podocytes in diabetic rats (59, 60). In addition, Etxeberria et al. observed that supplementation of the flavonol quercetin could inhibit the growth of *Erysipelotrichacea* (61). In these studies, dietary intake regulates and reshapes the composition of the gut microbiota, which is similar to our research. Our pumpkin polysaccharides could inhibit the growth of *Erysipelotrichaceae* to relieve the symptoms of diabetes. At present, there is limited evidence on the effect of pumpkin polysaccharides on the gut microbiota. There is only one study on the effect of pumpkin polysaccharides on gut microbiota, and it changed the structure of gut microbiota, mainly showing that it selectively enriches some key bacterial genera of *Bacteroidetes, Prevotella, Deltaproteobacteria, Oscillospira, Veillonellaceae, Phascolarctobacterium, Sutterella*, and *Bilophila* (7), which is similar to our result. These bacteria can produce butyric acid to promote insulin secretion after meals and improve diabetes (62). In short, our research shows that the treatment of pumpkin polysaccharides reshapes the intestinal flora of T2DM mice by increasing the abundance of intestinal flora, reducing the abundance of harmful bacteria, and promoting the growth of probiotics. Among them, the most representative ones are *Erysipelotrichaceae* and *Akkermansia*. The correlation between these gut microbiotas and the production of short-chain fatty acids, especially butyric acid, revealed the potential mechanism of pumpkin polysaccharides in the treatment of T2DM, and ultimately mediate their beneficial effects on the host.

In conclusion, the extraction and purification methods had a significant influence on extraction yield, polysaccharide content, physicochemical properties, monosaccharide composition, preliminary structural characterization, microstructure, and *in vitro* antioxidant and hypoglycemic activity. W-CCPs and RPPs had a significantly positive capacity to scavenge·OH, DPPH, ${\text{O}}_{2}^{-}$ and ABTS^+^ radicals, and α-glucosidase and α-amylase inhibitory activities. The RPPs had a strong glucose uptake and transport inhibition in the order of RPP-3 > RPP-4 > RPP-1 > RPP-2. RPP-3 could not only inhibit the uptake of glucose in the Caco-2 monolayer, but it could also promote the excretion of glucose. The animal experiment results indicate that treatment with crude pumpkin polysaccharides could significantly improve the symptoms of T2DM mice by lowering FBG, reducing insulin resistance, and lowering blood lipid TC, TG, and LDL levels. It could increase the diversity of intestinal flora and reduce the harmful flora of model mice. The pumpkin polysaccharides reshaped the gut microbiota by reducing *Erysipelotrichaceae* and increasing *Akkermansia* abundance, thereby improving the blood sugar and blood lipids of the T2DM mice.

## Data Availability Statement

The original contributions presented in the study are publicly available. This data can be found at: National Center for Biotechnology Information (NCBI) BioProject database under accession number PRJNA760239.

## Ethics Statement

The animal study was reviewed and approved by the Animal Center of Hubei University of Traditional Chinese Medicine.

## Author Contributions

FY, D-yL, and H-qW conceived and designed the experiments and wrote the article. FY, H-qW, and Z-lM performed the experiments. FY and H-qW analyzed the data. FY, H-qW, PW, Y-hG, and D-xZ contributed reagents, materials, and analysis tools. All authors have read and approved the recent form of this article.

## Funding

We gratefully acknowledge the financial support from the Hubei Provincial Central Government Guides Local Science and Technology Development Special Project (2020ZYYD008).

## Conflict of Interest

The authors declare that the research was conducted in the absence of any commercial or financial relationships that could be construed as a potential conflict of interest.

## Publisher's Note

All claims expressed in this article are solely those of the authors and do not necessarily represent those of their affiliated organizations, or those of the publisher, the editors and the reviewers. Any product that may be evaluated in this article, or claim that may be made by its manufacturer, is not guaranteed or endorsed by the publisher.
