# Supplementary material for: Sequential Extraction, Characterization, and Analysis of Pumpkin Polysaccharides for Their Hypoglycemic Activities and Effects on Gut Microbiota in Mice
Source: Front Nutr. 2021 Nov 3;8:769181. doi: 10.3389/fnut.2021.769181 (PMC8596442; doi:10.3389/fnut.2021.769181)
Supplement: Supplementary file 1 [file Data_Sheet_1.docx]

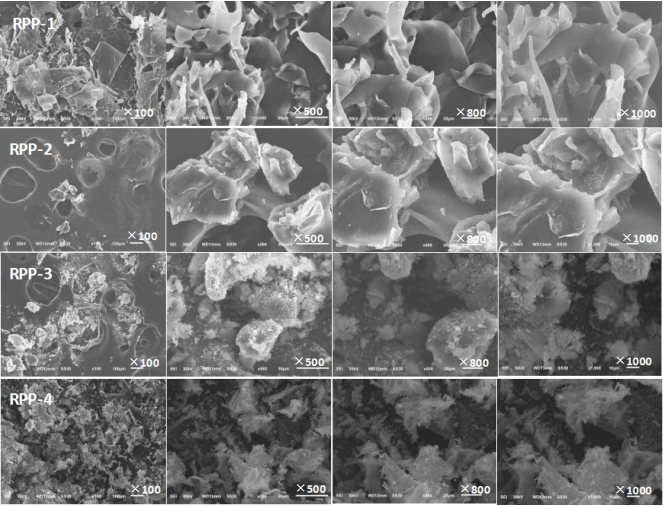


**Supplemental File 1** ﻿ SEM of RPPs. Magnifications from left to right: ×100 , ×500, ×800 , ×1000. RPP, refined pumpkin polysaccharides. Deproteinized pumpkin polysaccharides was separated in DEAE-52 cellulose gel column to obtain RPP-1, RPP-2, RPP-3, and RPP-4, of which, RPP-1 was a neutral polysaccharide, RPP-2, RPP-3, and RPP-4 were all acidic polysaccharides.


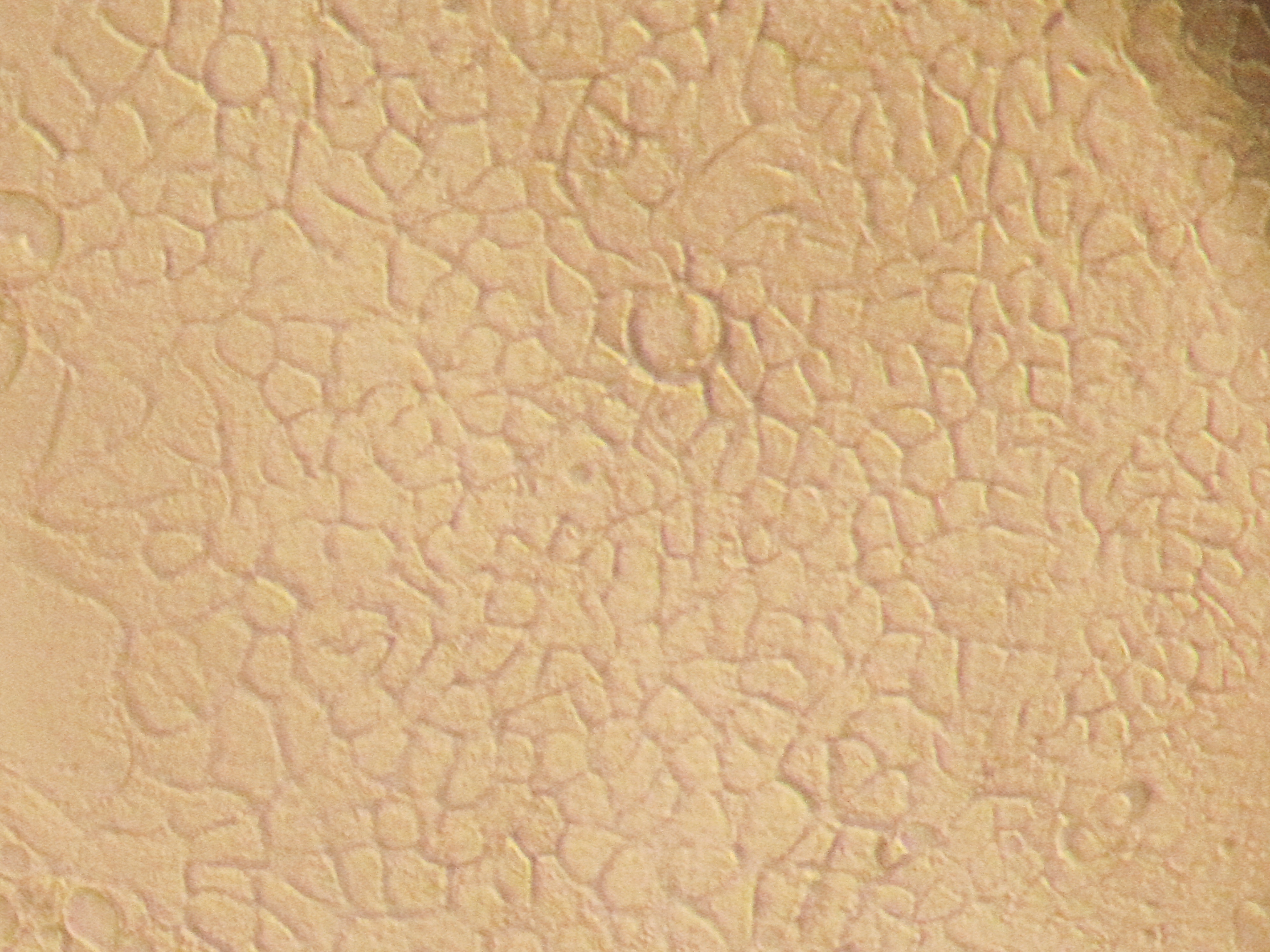

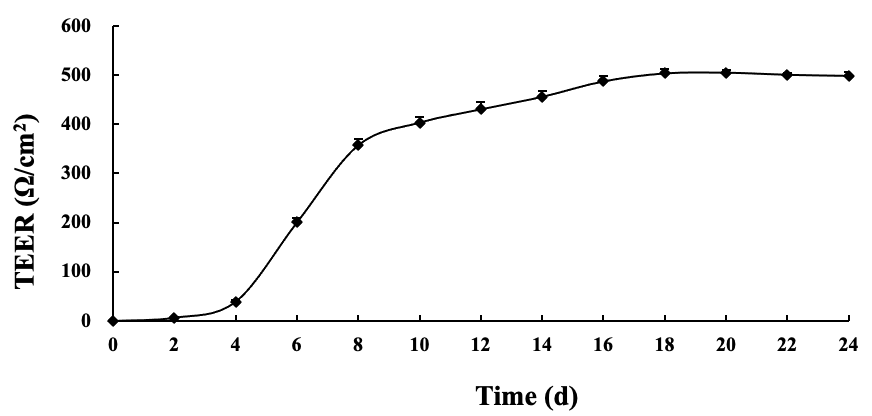


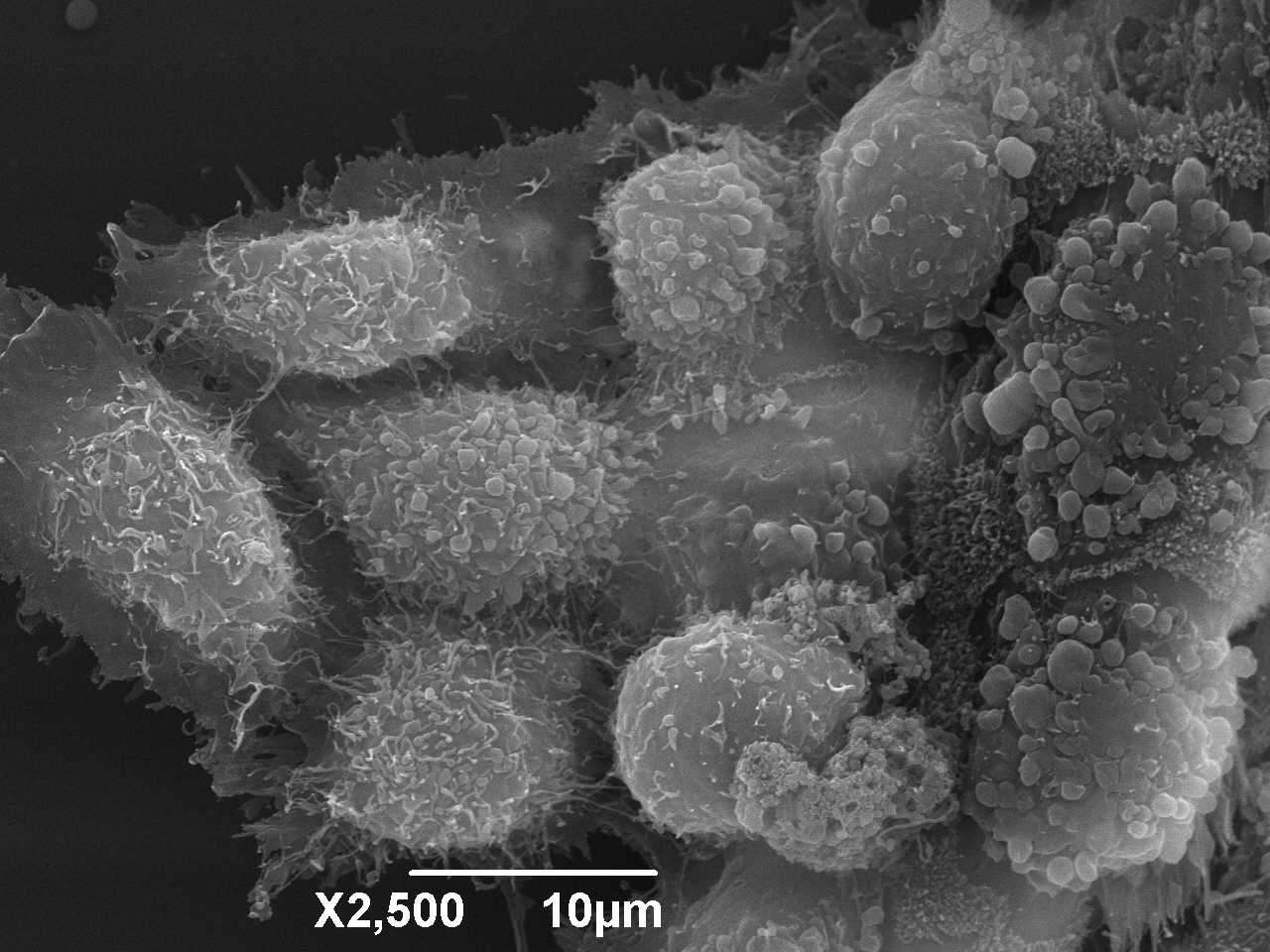

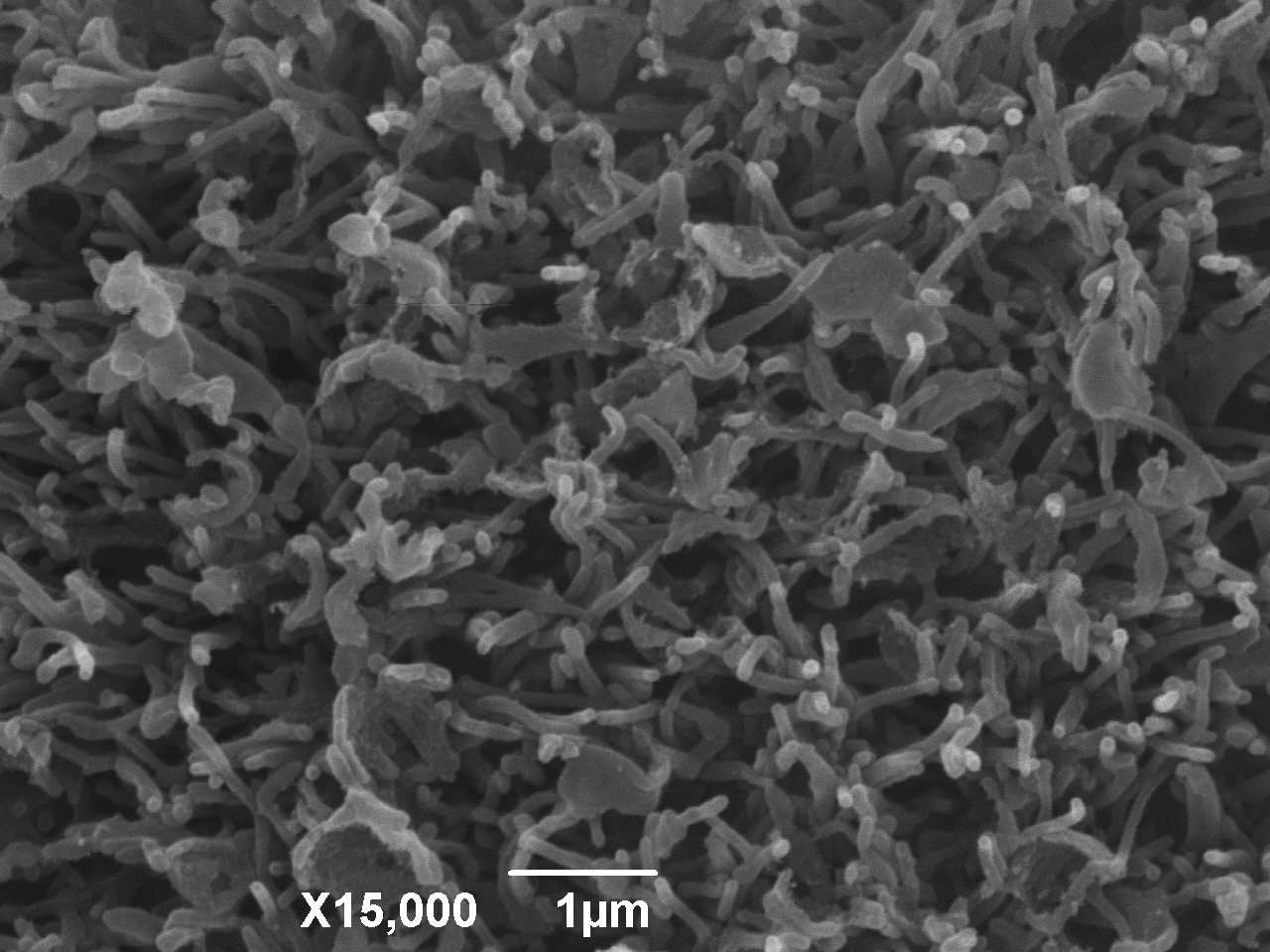


**D**

**C**







**F**

**E**

**Supplemental File 2** ﻿Morphology and characteristics of Caco-2 cell monolayer during and after differentiation. **(A)** Caco-2 monolayer morphology after the 21-day culture (Inverted microscope ×400). **(B)** Transepithelial electrical resistances (TEER) of Caco-2 monolayer at different time spots of the 24-day culture. **(C)** Morphology of Caco-2 monolayer (× 2500) under scanning electron microscope. **(D)** Microvilli on the apical side of the Caco-2 monolayer (× 15,000) under scanning electron microscope. **﻿(E and F)** The transmission electron micrographs of differentiated Caco-2 cells with microvilli (E: ×24,000) and tight junctions among cells (F: ×12,000) marked by the red arrow, respectively.
